# Synthesis of tetrasubstituted pyrazoles containing pyridinyl substituents

**DOI:** 10.3762/bjoc.13.90

**Published:** 2017-05-12

**Authors:** Josef Jansa, Ramona Schmidt, Ashenafi Damtew Mamuye, Laura Castoldi, Alexander Roller, Vittorio Pace, Wolfgang Holzer

**Affiliations:** 1 Division of Drug Synthesis, Department of Pharmaceutical Chemistry, Faculty of Life Sciences, University of Vienna, Althanstrasse 14, A-1090 Vienna, Austria; 2 Research Institute for Organic Syntheses (VUOS), Rybitví 296, CZ-533 54 Pardubice-Rybitví, Czech Republic; 3 Department of Organic Chemistry, Faculty of Science, Palacký University, 17. listopadu 1192/12, CZ-771 46 Olomouc, Czech Republic; 4 X-ray Structure Analysis Centre, Faculty of Chemistry, University of Vienna, Währinger Straße 42, A-1090 Vienna, Austria

**Keywords:** Negishi coupling, NMR (^1^H, ^13^C, ^15^N), pyrazole, pyridine, X-ray structure analysis

## Abstract

A synthesis of tetrasubstituted pyrazoles containing two, three or four pyridinyl substituents is described. Hence, the reaction of 1,3-dipyridinyl-1,3-propanediones with 2-hydrazinopyridine or phenylhydrazine, respectively, affords the corresponding 1,3,5-trisubstituted pyrazoles. Iodination at the 4-position of the pyrazole nucleus by treatment with I_2_/HIO_3_ gives the appropriate 4-iodopyrazoles which served as starting materials for different cross-coupling reactions. Finally, Negishi cross-coupling employing organozinc halides and Pd catalysts turned out to be the method of choice to obtain the desired tetrasubstituted pyrazoles. The formation of different unexpected reaction products is described. Detailed NMR spectroscopic investigations (^1^H, ^13^C, ^15^N) were undertaken with all products prepared. Moreover, the structure of a condensation product was confirmed by crystal structure analysis.

## Introduction

**Scheme 1:**
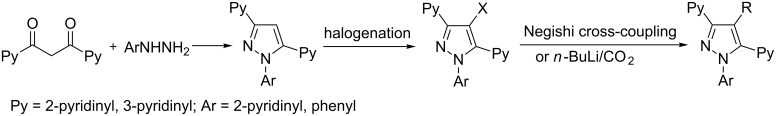
Envisaged general approach for the synthesis of the title compounds.

The pyrazole nucleus is a frequently occurring motif in many pharmaceuticals [1–2] and biologically active compounds [3–4], agrochemicals [5], dyes [6], fluorescent materials [7–8] and ligands of complexing agents [9–11]. Multiaryl-substituted pyrazoles are of special interest, with some drug molecules such as the nonsteroidal anti-inflammatory agent Lonazolac [12] or the well-known COX-2 inhibitor Celecoxib [13] as prominent representatives. Moreover, tetrasubstituted pyrazoles have shown to act, for instance, as estrogen receptor antagonists [14–15], endothelin antagonists [16], lipoxygenase inhibitors [17] and special luminophores [18]. For such fully substituted pyrazoles different synthetic approaches have been published. The most common strategies employ reactions of 1,3-dicarbonyl compounds or α,β-unsaturated carbonyl compounds with substituted hydrazines [4,6,19]. To overcome the drawbacks of this method, namely insufficient regioselectivity [20], other accesses such as, for instance, regioselective metalations of N-protected pyrazoles [21] or sequential cross-coupling reactions starting from 3-iodopyrazole [22] have been described. Herein, we report the synthesis of fully substituted pyrazoles containing at least two pyridinyl substituents by combining the before mentioned approaches: reaction of 1,3-dipyridinyl-1,3-diketones with arylhydrazines, halogenation of the resulting 1,3,5-triarylpyrazoles in the 4-position and further functionalization via Negishi cross-coupling [23–24] or halogen–lithium exchange reaction (Scheme 1). The resulting compounds amongst others seem to be interesting as potential complexing agents.

## Results and Discussion

### Chemistry

#### Synthesis of 4-iodopyrazoles **3a–d**

**Scheme 2:**
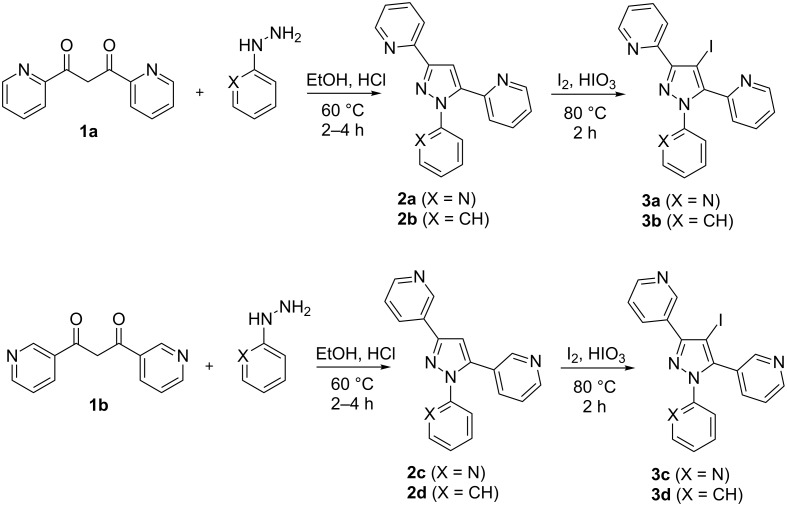
Synthesis of 4-iodopyrazoles of type **3**.

As starting materials the symmetrical 1,3-diketones **1a** and **1b** were employed, which were obtained by condensation of ethyl 2- or 3-pyridinecarboxylates with the appropriate 2- or 3-acetylpyridines following known procedures [25–26]. Reaction of **1a** and **1b** with 2-hydrazinopyridine and phenylhydrazine, respectively, afforded the tri(hetero)arylpyrazoles **2a–d** which were further converted into the corresponding 4-iodopyrazole derivatives **3a–d** by treatment with I_2_/HIO_3_ in acetic acid at 80 °C (Scheme 2). The latter iodination method turned out to be superior to the reaction of compounds **2** with *N*-iodosuccinimide. Species **3a–d** served as educts for the investigations concerning further functionalization at pyrazole C-4.

#### Carboxylation of 4-iodopyrazoles **3a–d**

**Scheme 3:**
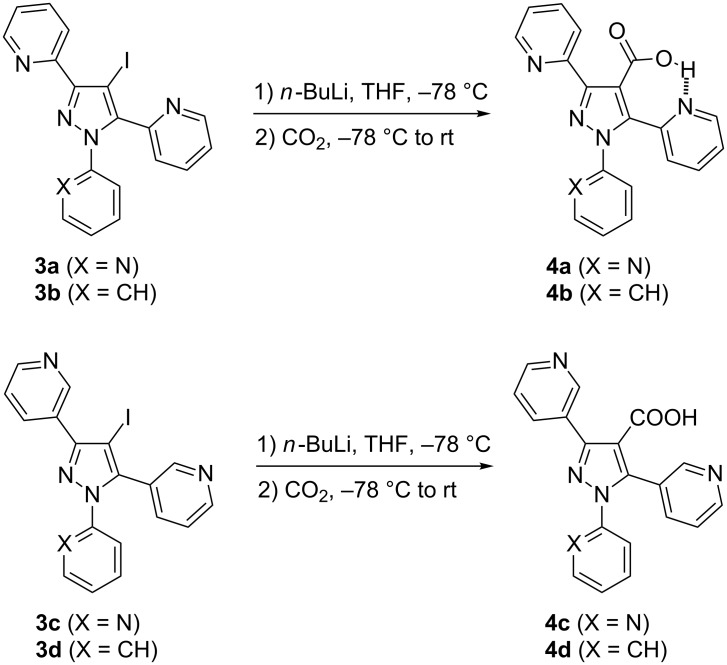
Lithium–halogen exchange and subsequent carboxylation with iodopyrazoles **3a–d**.

The lithium–iodine exchange proceeded quickly and quantitatively in case of 3,5-di(pyridin-2-yl)-substituted derivatives **3a**,**b** upon treatment with 1.1 equivalents of *n*-BuLi at −78 °C. Subsequent reaction with CO_2_ led to almost complete conversion to **4a**,**b** as detected by TLC (Scheme 3). In contrast, with 3,5-di(pyridin-3-yl)-substituted derivatives **3c**,**d**, the lithiation reaction was slower and not fully complete, also the subsequent reaction with CO_2_ was more sluggish in comparison to **3a**,**b** what resulted in lower yields. The increased reactivity of **3a**,**b** compared to **3c**,**d** may be explained by the ability of the former to stabilize the intermediate organolithium species by chelation due to the pyridine nitrogen atoms. The 4-pyrazolecarboxylates **4a**,**b** are capable to form intramolecular hydrogen bonds of the carboxylic OH proton with the neighbouring pyridine nitrogen atoms, which is manifested by large chemical shift values (≈18 ppm, in CDCl_3_) of the concerning OH proton in the ^1^H NMR spectra. The marked decrease of the ^15^N chemical shift of the nitrogen atom of the pyridine attached at pyrazole C-5 compared to those of the corresponding nitrogen atoms in compounds **2a**,**b** and **3a**,**b** (whereas the ^15^N shift of the pyridine moiety attached to pyrazole C-3 only slightly differs for compounds **2a**,**b**, **3a**,**b** and **4a**,**b**) strongly hints to the involvement of the former into an intramolecular hydrogen bond as indicated in Scheme 3.

#### Cross-coupling reactions

**Scheme 4:**
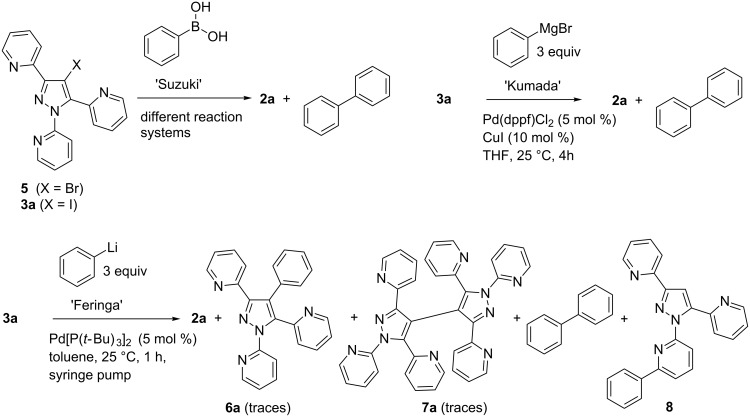
Attempted cross-coupling reactions with 4-halopyrazoles **5** and **3a**.

Initial attempts to react 4-bromopyrazole **5** – obtained from reaction of **2a** with *N*-bromosuccinimide – with phenylboronic acid (or 3-pyridineboronic acid, respectively) in the course of a Suzuki cross-coupling procedure [27–28] under different reaction conditions (for instance Cs_2_CO_3_/DMF; Na_2_CO_3_/DMF; Cs_2_CO_3_/dioxane–H_2_O; Na_2_CO_3_/dioxane–H_2_O; Pd(PPh_3_)_4_) always led to dehalogenation and thus to the isolation of **2a** as the main reaction product accompanied by some biphenyl (Scheme 4). As well, realizing the reaction under microwave assistance (150 °C, 800 W) led to the same result. Employing the corresponding iodo derivative **3a** as the starting material again resulted mainly in the formation of **2a** (4 equiv Na_2_CO_3_/EtOH–H_2_O 4:1/Pd(dppf)Cl_2_ 5 mol %, 24 h reflux, [29]) (Scheme 4). The reaction of **3a** with phenylmagnesium bromide under modified ‘Kumada’-conditions [30] (Scheme 4) once more gave **2a** (dehalogenation) and biphenyl.

Subjecting **3a** and phenyllithium under the conditions of a ‘Feringa’-coupling [31] resulted in a complex mixture with **2a** as the main product, but also containing biphenyl, traces of the desired product **6a**, the homocoupling dimer **7a** as well as a compound **8**, obviously resulting from attack of PhLi to the pyridine system attached to pyrazole N-1 (Scheme 4).

#### Negishi cross-couplings with 4-iodopyrazoles **3a–d**

**Scheme 5:**
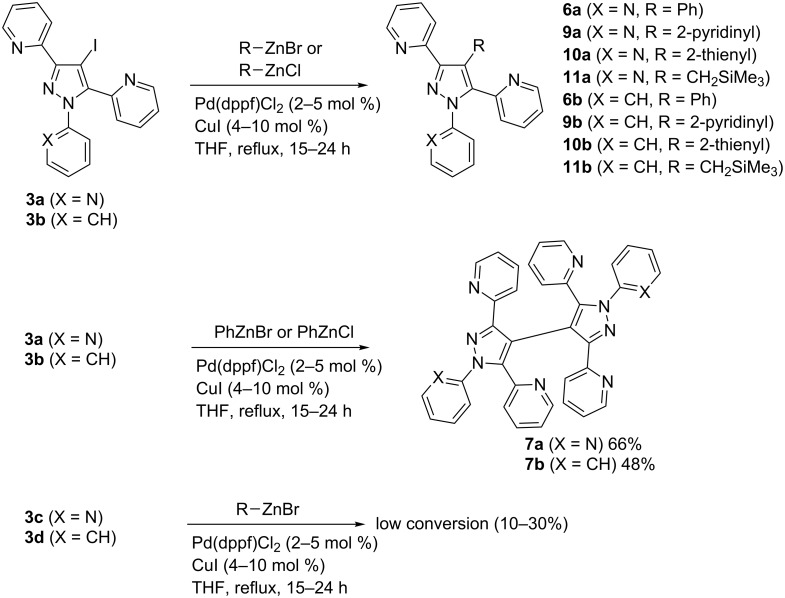
Negishi couplings with 4-iodopyrazoles **3a,b**.

The Negishi cross-coupling reaction with organozinc compounds [23–24] is a valuable tool for the formation of C–C bonds, particularly in the presence of functional groups. The employed organozinc reagents are relatively reactive nucleophiles undergoing rapid transmetalation with appropriate transition metal species, for instance palladium salts. This method has been successfully applied in the synthesis of bi(hetero)aryls [32–34] and thus also in those of C-substituted pyrazoles [35–36]. In our case, the reaction of iodopyrazoles **3a,b** with different organozinc derivatives (Scheme 5) proceeded smoothly leading to the coupling products **6a,b** and **9a,b**–**11a,b** in variable yields (19–87%). Firstly, the Pd(PPh_3_)_4_ was employed as the catalyst for the reaction of **3a** to **9a** but low conversion and considerable dehalogenation were observed. Utilization of Pd(dppf)Cl_2_ as catalyst in THF brought much better selectivity, because dehalogenation was practically not observed, but the conversion was still unsatisfying. Despite of these results, cocatalysis with CuI [37] was tested and showed a significant rate-enhancing effect and low portion of dehalogenation. Interestingly, the lowest yields were obtained with phenylzinc halides, here the homocoupling products **7a**,**b** emerged as the predominant reaction products (Scheme 5, middle trace), probably due to preferable halogen–zinc exchange and subsequent homocoupling. Interestingly, such sterically hindered halides provided homocoupling products **7a**,**b** in good yields (66% and 48%, respectively). The selectivity towards these compounds increased with the excess of phenylzinc halide. In a model reaction employing **3b** and 2 equivalents of PhZnBr, a mixture of **7b** (48%), the desired coupling product **6b** (27%) and dehalogenation product **2b** (25%) was isolated. Such homocoupling and dehalogenation processes have been also observed in the course of related Pd-catalyzed cross-coupling reactions, such as, for instance, in Suzuki–Miyaura reactions [38]. In contrast, the corresponding 2-pyridinyl or 2-thienyl organozinc congeners gave much better results with nearly no homocoupling side reactions and only few amounts of dehalogenation products observed. The reactions were carried out either using 2 equivalents of commercially available RZnBr or 3 equivalents of in-situ-prepared RZnCl (see Supporting Information File 1). Similarly as observed in the course of the lithiation/carboxylation reactions (Scheme 3), low reactivity was detected with precursors **3c**,**d** resulting in conversion rates below 30% (Scheme 5, lowest trace).

**Scheme 6:**
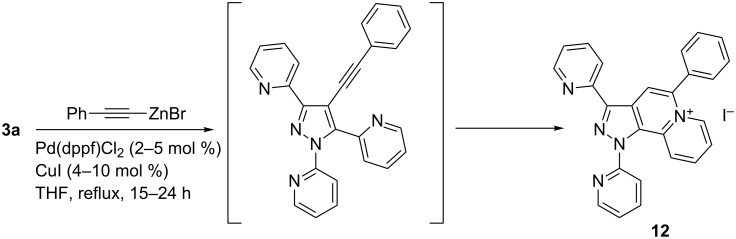
Formation of pyrazoloquinolizin-6-ium iodide **12** upon reaction of **3a** with (phenylethynyl)zinc bromide.

An interesting compound was isolated in low yield (≈14%) from the mixture obtained upon the reaction of **3a** with (phenylethynyl)zinc bromide. By careful NMR and HRMS analysis the pyrazolo[3,4-*a*]quinolizin-6-ium structure **12** was established, which was confirmed by single crystal X-ray structure analysis (see Supporting Information File 1), proving also iodide as the corresponding anion [39] which acts as a leaving group in the coupling reaction and also could origin from the CuI co-catalyst. A possible explanation for the formation of the condensation product **12** consists in intramolecular cyclization of the intermediate 4-alkynylpyrazole under the reaction conditions (Scheme 6).

### NMR spectroscopic investigations

In Supporting Information File 1 the NMR spectroscopic data of all compounds treated within this study are indicated. Full and unambiguous assignment of ^1^H, ^13^C and nearly all ^15^N NMR resonances was achieved by combining standard NMR techniques [40], such as fully ^1^H-coupled ^13^C NMR spectra, APT, gs-HSQC, gs-HMBC, gs-HSQC-TOCSY, COSY, TOCSY and NOESY spectroscopy.

**Scheme 7:**
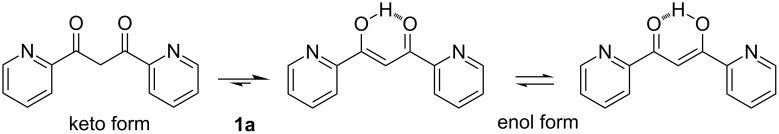
Prototropic tautomerism of compound **1a**.

The ^1^H and ^13^C NMR spectra (in CDCl_3_ solution) of precursors **1** denote these compounds to be predominantly present in the enol form (Scheme 7). This is in contrast to ref. [25], where only traces of the enol form were detected for **1a**, however, by recordings in acetone-*d*_6_ solution. Whereas for **1a** the enol/keto ratio is ≈6.6:1, for the 3-pyridinyl congener **1b** it was found to be 187:1. Switching to the more polar solvent DMSO-*d*_6_ enhances the amount of the keto form to ≈3% (ratio 35:1). The enol forms of compounds **1** show the characteristically large chemical shifts for the OH proton (**1a**: 15.94 ppm, **1b**: 16.52 ppm, in CDCl_3_).

4-Iodopyrazoles **3a–d** are characterized by a marked upfield shift of the signal due to pyrazole C-4 compared the corresponding precursors **2a–d** as a result of the heavy-atom α-effect of the iodine atom.

The different hydrogen bonding situation in carboxylic acids **4a**–**d** and the resultant consequences for the concerned ^15^N NMR chemicals shifts have been already mentioned before (see also Scheme 3).

For all compounds carrying several pyridine moieties in the same molecule the individual azine systems could be unambiguously discriminated. Expectedly, in all cases the ^15^N NMR chemical shifts of the pyridine nitrogens of the azine ring attached to pyrazole N-1 are somewhat smaller than those of the corresponding pyridine N-atoms in systems attached to pyrazole C-3, C-5 (or C-4), whereas the C-3-pyridine nitrogen atom in many cases was the most deshielded one. The pyridinium-type nitrogen (N-6) in condensed system **12** shows a characteristic upfield shift (δ_N-6_ −176.2 ppm) compared to the ‘usual’ ^15^N NMR chemical shifts of 2-pyridinyl systems attached to pyrazole C-5 (δ ≈ −62 to −70 ppm). This is in perfect agreement with the ≈100 ppm upfield shift observed when switching from pyridine to pyridine hydrochloride [41–42].

Other interesting phenomena are the unusually large ^1^H NMR chemical shifts for protons H-3 (and more rare H-5) at the pyridine rings (in pyridine itself δ_H-3/5_ is ≈7.4ppm). Thus, for instance, in **11a** H-3 of C-3 pyridine shows a chemical shift of 8.09 ppm (whereas the corresponding H-5 has only 7.22 ppm). A possible explanation is the proximity to nitrogen lone-pairs of pyrazole N-2 (or adjacent pyridine-N lone-pairs in suitable conformations) which affect the mentioned protons by electrostatic field effects resulting in markedly higher chemical shifts – as shown, for instance for 2,2’-bipyridines [43–44] and other comparable systems [45].

**Figure 1:**
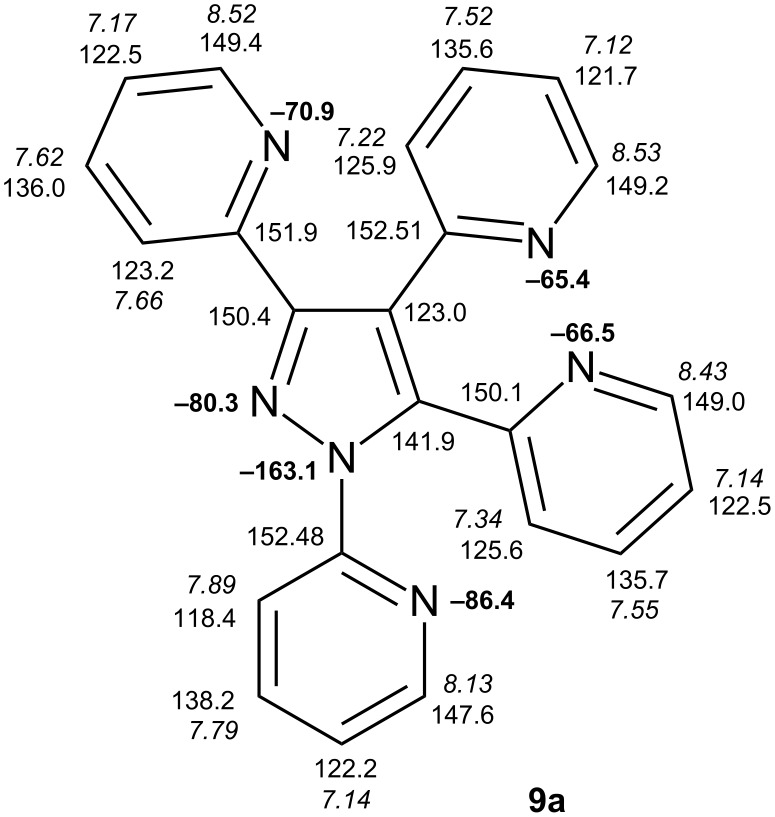
^1^H NMR (in italics), ^13^C NMR and ^15^N NMR (in bold) chemical shifts of compound **9a** (in CDCl_3_).

In Figure 1, complete NMR data for the tetrapyridinylpyrazole **9a** are displayed, which permits to follow some of the trends regarding chemical shift considerations discussed above.

## Conclusion

Pd-catalyzed cross-coupling reactions of iodopyrazoles **3a**,**b** under standard conditions are characterized by the occurrence of different (unwanted) products resulting mainly from dehalogenation and homocoupling processes. Nevertheless, the Negishi coupling here seems to be the method of choice for C–C bond formations at pyrazole C-4. In contrast, 3-pyridinyl congeners **3c**,**d** gave markedly lower conversion rates in all reactions investigated.

## Supporting Information

File 1 Experimental details and characterization data.
