# Supplementary material for: Synthesis of tetrasubstituted pyrazoles containing pyridinyl substituents
Source: Beilstein J Org Chem. 2017 May 12;13:895–902. doi: 10.3762/bjoc.13.90 (PMC5433223; doi:10.3762/bjoc.13.90)
Supplement: File 1 — Experimental details and characterization data. [file Beilstein_J_Org_Chem-13-895-s001.pdf]

Supporting Information

for

Synthesis of tetrasubstituted pyrazoles containing pyridinyl substituents

Josef Jansa[1,2,3], Ramona Schmidt[1], Ashenafi Damtew Mamuye[1], Laura Castoldi[1], Alexander Roller[4], Vittorio Pace[1] and Wolfgang Holzer*[1]

Address: [1]Division of Drug Synthesis, Department of Pharmaceutical Chemistry, Faculty of Life Sciences, University of Vienna, Althanstrasse 14, A-1090 Vienna, Austria, [2]Research Institute for Organic Syntheses (VUOS), Rybitví 296, CZ-533 54 Pardubice-Rybitví, Czech Republic, [3]Department of Organic Chemistry, Faculty of Science, Palacký University, 17. listopadu 1192/12, CZ-771 46 Olomouc, Czech Republic and [4]X-ray Structure Analysis Centre, Faculty of Chemistry, University of Vienna, Währinger Straße 42, A-1090 Vienna, Austria

Email: Wolfgang Holzer - wolfgang.holzer@univie.ac.at

*Corresponding author

Experimental details and characterization data

1. General

Melting points were determined on a Reichert–Kofler hot-stage microscope and are uncorrected. Mass spectra were obtained on a Shimadzu QP 1000 instrument (EI, 70 eV) and on a Bruker maXis 4G instrument (ESI–TOF, HRMS). ^{1}H, ^{13}C and ^{15}N NMR spectra were recorded with a Bruker Avance III 400 spectrometer (400 MHz for ^{1}H, 100 MHz for ^{13}C, 40 MHz for ^{15}N) or a Bruker Avance 500 spectrometer (500 MHz for ^{1}H, 125 MHz for ^{13}C, 50 MHz for ^{15}N) at 297 K using "directly" detecting broadband observe (BBFO) probes. The center of the solvent signal was used as an internal standard which was related to TMS with δ 7.26 ppm (^{1}H in $CDCl_3$) and δ 77.0 ppm (^{13}C in $CDCl_3$). ^{15}N NMR spectra (gs-HMBC) were referenced against neat, external nitromethane. Digital resolutions were 0.2 Hz/data point in the ^{1}H NMR spectra and 0.3 Hz/data point in the ^{13}C NMR spectra. For column chromatographic separations Merck Kieselgel 60 (70–230 mesh) was used. Light petroleum refers to the fraction with boiling point 40–65 °C. Elemental analyses were performed at the Microanalytical Laboratory, University of Vienna. Yields are not optimized.

2. (2*Z*)-3-Hydroxy-1,3-di(2-pyridinyl)-2-propen-1-one (enol form) and tautomeric 1,3-di(2-pyridinyl)-1,3-propanedione (keto form) (**1a**)

Under argon, to a suspension of sodium hydride (60% dispersion in mineral oil, 2.0 g, 50 mmol) in dry THF (200 ml) 2-acetylpyridine (5.0 g, 41.3 mmol) and ethyl 2-pyridinecarboxylate (6.24 g, 41.3 mmol) were added. The reaction mixture was slowly heated to 60 °C in the course of 1 h and then kept at this temperature for 15 h. During this time, a fine yellow suspension was formed. The mixture was cooled to 0–5 °C, filtered off, washed with THF (50 ml) and dried to afford the sodium salt of **1a** as a beige powder. Then this material was suspended in water (300 ml) and acetic acid (3.2 ml, 55.9 mmol) was slowly added with stirring. The thick suspension formed was filtered off, the remaining product was washed with water (150 ml), and dried to afford 7.10 g (76%) of an off-white solid: mp 100 °C (lit. [1] mp 100-101 °C). In $CDCl_3$ solution, **1a** was present as a mixture of the enol and the keto form in a ratio of ≈6.6 :1. Enol form: ^{1}H NMR ($CDCl_3$, 400 MHz): δ (ppm) 15.94 (br s, 1H, OH), 8.73 (ddd, $^3J_{6,5}$ = 4.7 Hz, $^4J_{6,4}$ = 1.8 Hz, $^5J_{6,3}$ = 0.9 Hz, 2H, Pyrid H-6), 8.13 (ddd, $^3J_{3,4}$ = 7.9 Hz, $^4J_{3,5}$ = 1.2 Hz, $^5J_{3,6}$ = 0.9 Hz, 2H, Pyrid H-3), 8.12 (s, 1H, =CH), 7.84 (ddd, $^3J_{4,3}$ = 7.9 Hz, $^3J_{4,5}$ = 7.6 Hz, $^4J_{4,6}$ = 1.8 Hz, 2H, Pyrid H-4), 7.42 (ddd, $^3J_{5,4}$ = 7.6 Hz, $^3J_{5,6}$ = 4.7 Hz, $^4J_{5,3}$ = 1.2 Hz, 2H, Pyrid H-5). ^{13}C NMR ($CDCl_3$, 100 MHz): δ (ppm) 184.4 (C-1, C-3), 152.3 (Pyrid C-2), 149.4 (Pyrid C-6), 136.80 (Pyrid C-4), 126.3 (Pyrid C-5), 122.0 (Py C-3), 94.5 (C-2). ^{15}N NMR ($CDCl_3$, 40 MHz): δ (ppm) –70.1 (Pyrid N-1). Keto form: ^{1}H NMR ($CDCl_3$, 400 MHz): δ (ppm) 8.57 (ddd, $^3J_{6,5}$ = 4.7 Hz, $^4J_{6,4}$ = 1.8 Hz, $^5J_{6,3}$ = 1.0 Hz, 2H, Pyrid H-6), 8.07 (ddd, $^3J_{3,4}$ = 7.8 Hz, $^4J_{3,5}$ = 1.2 Hz, $^5J_{3,6}$ = 1.0 Hz, 2H, Pyrid H-3), 7.82 (ddd, $^3J_{4,3}$ = 7.8 Hz, $^3J_{4,5}$ = 7.6 Hz, $^4J_{4,6}$ = 1.8 Hz, 2H, Pyrid H-4), 7.42 (ddd, $^3J_{5,4}$ = 7.6 Hz, $^3J_{5,6}$ = 4.7 Hz, $^4J_{5,3}$ = 1.2 Hz, 2H, Pyrid H-5), 4.93 (s, 2H, CH_2). ^{13}C NMR ($CDCl_3$, 100 MHz): δ (ppm) 196.9 (C=O), 152.6 (Pyrid C-2), 148.8 (Pyrid C-6), 136.84 (Pyrid C-4), 127.1 (Pyrid C-5), 121.9 (Pyrid C-3), 48.2 (CH_2). ^{15}N NMR ($CDCl_3$, 40 MHz): δ (ppm) –67.6 (Pyrid N-1).

3. (2*Z*)-3-Hydroxy-1,3-di(3-pyridinyl)-2-propen-1-one (enol form) (**1b**)

Starting from 3-acetylpyridine (5.0 g, 41.3 mmol) and ethyl nicotinate (6.24 g, 41.3 mmol) compound **1b** was prepared similarly to the procedure described for the synthesis of **1a**. Recrystallization from EtOH gave 7.00 g (75%) of yellowish crystals: mp 192-193 °C (lit. [2] mp 199-201 °C). The NMR spectra in $CDCl_3$ solution showed almost exclusively the enol form, whereas the keto form was present only to ≈0.5 %. Enol form: ^{1}H NMR ($CDCl_3$, 400 MHz): δ (ppm) 16.52 (br s, 1H, OH), 9.19 (dd, $^4J_{2,4}$ = 2.3 Hz, $^5J_{2,5}$ = 0.8 Hz, 2H, Pyrid H-2), 8.78 (dd, $^3J_{6,5}$ = 4.8 Hz, $^4J_{6,4}$ = 1.7 Hz, 2H, Pyrid H-6), 8.26 (ddd, $^3J_{4,5}$ = 8.0 Hz, $^4J_{4,2}$ = 2.3 Hz, $^4J_{4,6}$ = 1.7 Hz, 2H, Pyrid H-4), 7.45 (ddd, $^3J_{5,4}$ = 8.0 Hz, $^3J_{5,6}$ = 4.8 Hz, $^5J_{5,2}$ = 0.8 Hz, 2H, Pyrid H-5), 6.86 (s, 1H, =CH). ^{13}C NMR ($CDCl_3$, 100 MHz): δ (ppm) 184.1 (C-1, C-3), 153.2 (Pyrid C-6), 148.5 (Pyrid C-2), 134.6 (Pyrid C-4), 130.8 (Pyrid C-3), 123.6 (Pyrid C-5), 93.7 (C-2). ^{15}N NMR ($CDCl_3$, 40 MHz): δ (ppm) –68.9 (Pyrid N-1). EIMS, *m/z* (rel. int.): 227 (6) $[M+H]^+$, 226 (48) $[M]^+$, 225 (100) $[M-H]^+$, 197 (23), 148 (79), 106 (54), 79 (49), 78 (86), 69 (45), 65 (23), 52 (20), 51 (63), 50 (21). HRMS (ESI), *m/z*: calcd. for $C_{13}H_{11}N_2O_2^+$ 227.0815 $[M+H]^+$; found 227.0814.

4. General procedure for the preparation of pyrazoles **2a–d**

The 1,3-dielectrophile **1a** or **1b** (2.00 g, 8.84 mmol) was added to the solution of the corresponding hydrazine (2-hydrazinopyridine, phenylhydrazine, 8.84 mmol) in ethanol (60 ml). Then, 37% hydrochloric acid (0.6 ml, 7.3 mmol) was added and the suspension was heated under stirring at 60 °C for 2–4 h to obtain complete conversion, detected by TLC. The volatile matter was distilled off. Water (40 ml) and saturated solution of $NaHCO_3$ (20 ml) were added to the residue and the product was exhaustively extracted with CH_2Cl_2 (50 ml). The organic layer was washed with water (40 ml) and dried over anhydrous Na_2SO_4. Products **2a–d** were obtained after filtration and evaporation of the solvent and their purity was sufficient for further use. Analytical samples were obtained by the methods described below.

4.1. 2,2',2"-(1*H*-Pyrazole-1,3,5-triyl)tripyridine (**2a**)

Yield: 2.59 g (98%) of yellowish solid, pure according to NMR analysis. An analytical sample was obtained by column chromatography (SiO_2, ethyl acetate); mp 88-89 °C. ^{1}H NMR ($CDCl_3$, 400 MHz): δ (ppm) 8.66 (ddd, $^3J_{6,5}$ = 4.9 Hz, $^4J_{6,4}$ = 1.8 Hz, $^5J_{6,3}$ = 1.0 Hz, 1H, 3-Pyrid H-6), 8.45 (ddd, $^3J_{6,5}$ = 4.8 Hz, $^4J_{6,4}$ = 1.8 Hz, $^5J_{6,3}$ = 1.0 Hz, 1H, 5-Pyrid H-6), 8.26 (ddd, $^3J_{6,5}$ = 4.9 Hz, $^4J_{6,4}$ = 1.8 Hz, $^5J_{6,3}$ = 0.9 Hz, 1H, 1-Pyrid H-6), 8.12 (m, 1H, 3-Pyrid H-3), 7.82 (m, 1H, 1-Pyrid H-4), 7.77 (m, 1H, 1-Pyrid H-3), 7.74 (m, 1H, 3-Pyrid H-4), 7.71 (m, 1H, 5-Pyrid H-4), 7.54 (m, 1H, 5-Pyrid H-3), 7.37 (s, 1H, H-4), 7.24 (m, 1H, 3-Pyrid H-5), 7.21 (m, 1H, 1-Pyrid H-5), 7.19 (m, 1H, 5-Pyrid H-5). ^{13}C NMR ($CDCl_3$, 100 MHz): δ (ppm) 152.8 (1-Pyrid C-2), 152.6 (Pyrazole C-3), 151.6 (3-Pyrid C-2), 150.1 (5-Pyrid C-2), 149.4 (3-Pyrid C-6), 149.1 (5-Pyrid C-6), 147.9 (1-Pyrid C-6), 144.1 (Pyrazole C-5), 138.2 (1-Pyrid C-4), 136.5 (3-Pyrid C-4), 136.3 (5-Pyrid C-4), 123.3 (5-Pyrid C-3), 122.9 (3-Pyrid C-5), 122.6 (5-Pyrid C-5), 122.5 (1-Pyrid C-5), 120.4 (3-Pyrid C-3), 118.8 (1-Pyrid C-3), 108.1 (Pyrazole C-4). ^{15}N NMR ($CDCl_3$, 40 MHz): δ (ppm) –70.2 (5-Pyrid N-1), –77.4 (Pyrazole N-2), –77.8 (3-Pyrid N-1), –85.4 (1-Pyrid N-1), –163.6 (Pyrazole N-1). EIMS, *m/z* (rel. int.): 299 (50) $[M]^+$, 298 (100) $[M-H]^+$, 270 (14), 221 (24), 78 (28). HRMS (ESI), *m/z*: calcd. for $C_{18}H_{14}N_5^+$ 300.1244 $[M+H]^+$; found 300.1247. Anal. Calcd. for $C_{18}H_{13}N_5$ (299.33): C 72.23; H 4.38; N 23.40. Found; C 72.24; H 4.18; N 23.23.

4.2. 2,2'-(1-phenyl-1*H*-pyrazole-3,5-diyl)dipyridine (**2b**)

Yield: 2.56 g (97%) of a yellowish solid, pure according to NMR analysis. An analytical sample was obtained by column chromatography (SiO_2, ethyl acetate); mp 111-112 °C (lit. [3] mp 119 °C). ^{1}H NMR ($CDCl_3$, 400 MHz): δ (ppm) 8.67 (ddd, $^3J_{6,5}$ = 4.9 Hz, $^4J_{6,4}$ = 1.8 Hz, $^5J_{6,3}$ = 1.0 Hz, 1H, 3-Pyrid H-6), 8.55 (ddd, $^3J_{6,5}$ = 4.9 Hz, $^4J_{6,4}$ = 1.8 Hz, $^5J_{6,3}$ = 1.0 Hz, 1H, 5-Pyrid H-6), 8.09 (ddd, $^3J_{3,4}$ = 8.0 Hz, $^4J_{3,5}$ = 1.2 Hz, $^5J_{3,6}$ = 1.0 Hz, 1H, 3-Pyrid H-3), 7.74 (ddd, $^3J_{4,3}$ = 8.0 Hz, $^3J_{4,5}$ = 7.5 Hz, $^4J_{4,6}$ = 1.8 Hz, 1H, 3-Pyrid H-4), 7.64 (ddd, $^3J_{4,3}$ = 7.9 Hz, $^3J_{4,5}$ = 7.6 Hz, $^4J_{4,6}$ = 1.8 Hz, 1H, 5-Pyrid H-4), 7.41 (s, Pyrazole H-4), 7.39 (m, 2H, 1-Ph H-2,6), 7.37 (m, 2H, 1-Ph H-3,5), 7.35 (m, 1-Ph H-4), 7.33 (ddd, $^3J_{3,4}$ = 7.9 Hz, $^4J_{3,5}$ =

1.2 Hz, $^{5}J_{3,6}$ = 1.0 Hz, 1H, 5-Pyrid H-3), 7.23 (ddd, $^{3}J_{5,4}$ = 7.5 Hz, $^{3}J_{5,6}$ = 4.9 Hz, $^{4}J_{5,3}$ = 1.2 Hz, 1H, 3-Pyrid H-5), 7.20 (ddd, $^{3}J_{5,4}$ = 7.6 Hz, $^{3}J_{5,6}$ = 4.9 Hz, $^{4}J_{5,3}$ = 1.2 Hz, 1H, 5-Pyrid H-5). ^{13}C NMR ($CDCl_3$, 100 MHz): δ (ppm) 152.2 (Pyrazole C-3), 151.8 (3-Pyrid C-2), 149.6 (5-Pyrid C6), 149.49 (5-Pyrid C-2), 149.47 (3-Pyrid C-6), 143.7 (Pyrazole C-5), 140.5 (1-Ph C-1), 136.5 (3-Pyrid C4), 136.3 (5-Pyrid C-4), 128.8 (1-Ph C-3,5), 127.7 (1-Ph C-4), 125.4 (1-Ph C-2,6), 123.5 (5-Pyrid C-3), 122.74 (5-Pyrid C-5), 122.68 (3-Pyrid C-5), 120.3 (3-Pyrid C-3), 107.4 (Pyrazole C-4). ^{15}N NMR ($CDCl_3$, 40 MHz): δ (ppm) –69.3 (5-Pyrid N-1), –73.5 (Pyrazole N-2), –78.0 (3-Pyrid N-1), –167.3 (Pyrazole N-1). EIMS, *m/z* (rel. int.): 299 (6) $[M+H]^+$, 298 (38) $[M]^+$, 297 (100) $[M-H]^+$, 78 (15), 77 (11), 51 (18). HRMS (ESI), *m/z*: calcd. for $C_{19}H_{15}N_4^+$ 299.1291 $[M+H]^+$; found 299.1291.

4.3. 2-[3,5-Di(pyridin-3-yl)-1*H*-pyrazol-1-yl]pyridine (**2c**)

Yield 2.33 g (88%) of a yellowish solid, pure according to NMR analysis, mp 92 °C. ^{1}H NMR ($CDCl_3$, 400 MHz): δ (ppm) 9.16 (dd, $^{4}J_{2,4}$ = 2.2 Hz, $^{5}J_{2,5}$ = 0.9 Hz, 1H, 3-Pyrid H-2), 8.62 (dd, $^{3}J_{6,5}$ = 4.8 Hz, $^{4}J_{6,4}$ = 1.7 Hz, 1H, 3-Pyrid H-6), 8.61 (dd, $^{4}J_{2,4}$ = 2.2 Hz, $^{5}J_{2,5}$ = 0.9 Hz, 1H, 5-Pyrid H-2), 8.58 (dd, $^{3}J_{6,5}$ = 4.9 Hz, $^{4}J_{6,4}$ = 1.7 Hz, 1H, 5-Pyrid H-6), 8.28 (ddd, $^{3}J_{6,5}$ = 4.9 Hz, $^{4}J_{6,4}$ = 1.9 Hz, $^{5}J_{6,3}$ = 0.8 Hz, 1H, 1-Pyrid H-6), 8.25 (ddd, $^{3}J_{4,5}$ = 7.9 Hz, $^{4}J_{4,2}$ = 2.2 Hz, $^{4}J_{4,6}$ = 1.7 Hz, 1H, 3-Pyrid H-4), 7.86 (ddd, $^{3}J_{4,3}$ = 8.1 Hz, $^{3}J_{4,5}$ = 7.0 Hz, $^{4}J_{4,6}$ = 1.9 Hz, 1H, 1-Pyrid H-4), 7.82 (ddd, $^{3}J_{3,4}$ = 8.1 Hz, $^{4}J_{3,5}$ = 1.5 Hz, $^{5}J_{3,6}$ = 0.8 Hz, 1H, 1-Pyrid H-3), 7.69 (ddd, $^{3}J_{4,5}$ = 7.9 Hz, $^{4}J_{4,2}$ = 2.2 Hz, $^{4}J_{4,6}$ = 1.7 Hz, 1H, 5-Pyrid H-4), 7.39 (ddd, $^{3}J_{5,4}$ = 7.9 Hz, $^{3}J_{5,6}$ = 4.8 Hz, $^{5}J_{5,2}$ = 0.9 Hz, 1H, 3-Pyrid H-5), 7.31 (ddd, $^{3}J_{5,4}$ = 7.9 Hz, $^{3}J_{5,6}$ = 4.9 Hz, $^{5}J_{5,2}$ = 0.9 Hz, 1H, 5-Pyrid H-5), 7.25 (ddd, $^{3}J_{5,4}$ = 7.0 Hz, $^{3}J_{5,6}$ = 4.9 Hz, $^{4}J_{5,6}$ = 1.5 Hz, 1H, 1-Pyrid H-5), 6.91 (s, 1H, Pyrazole H-4). ^{13}C NMR ($CDCl_3$, 100 MHz): δ (ppm) 152.2 (1-Pyrid C-2), 149.8 (Pyrazole C-3), 149.5 (3-Pyrid C-6), 149.4 (5-Pyrid C-2), 149.3 (5-Pyrid C-6), 148.1 (1-Pyrid C-6), 147.4 (3-Pyrid C-2), 142.1 (Pyrazole C-5), 138.7 (1-Pyrid C-4), 136.1 (5-Pyrid C-4), 133.1 (3-Pyrid C-4), 128.4 (3-Pyrid C-3), 127.4 (5-Pyrid C-3), 123.6 (3-Pyrid C-5), 122.9 (5-Pyrid C-5), 122.7 (1-Pyrid C-5), 117.9 (1-Pyrid C-3), 107.3 (Pyrazole C-4). ^{15}N NMR ($CDCl_3$, 40 MHz): δ (ppm) –70.2 (3-Pyrid N-1), –70.8 (5-Pyrid N-1), –80.3 (Pyrazole N-2), –87.3 (1-Pyrid N-1), –163.8 (Pyrazole N-1). EIMS, *m/z* (rel. int.): 300 (4) $[M+H]^+$, 299 (30) $[M]^+$, 298 (100) $[M-H]^+$, 97 (21), 78 (28), 71 (28), 59 (33), 57 (38), 55 (25), 51 (25), 43 (54), 41 (22). HRMS (ESI), *m/z*: calcd. for $C_{18}H_{14}N_5^+$ 300.1244 $[M+H]^+$; found 300.1246.

4.4. (3,3'-(1-Phenyl-1*H*-pyrazole-3,5-diyl)dipyridine (**2d**)

Yield 2.56 g (97 %) of a yellowish solid, pure according to NMR analysis. An analytical sample was obtained by column chromatography (SiO_2, EtOAc/acetone 1:1); mp 111 °C (lit. [2] mp 121.5-122.5 °C). ^{1}H NMR ($CDCl_3$, 400 MHz): δ (ppm) 9.13 (br s, 1H, 3-Pyrid H-2), 8.60 (m, 2H, 3-Pyrid H-6, 5-Pyrid H-2), 8.57 (dd, $^{3}J_{6,5}$ = 4.9 Hz, $^{4}J_{6,4}$ = 1.7 Hz, 1H, 5-Pyrid H-6), 8.24 (ddd, $^{3}J_{4,5}$ = 7.9 Hz, $^{4}J_{4,2}$= 2.3 Hz und $^{4}J_{4,6}$ = 1.7 Hz, 1H, 3-Pyrid H-4), 7.51 (ddd, $^{3}J_{4,5}$ = 7.9 Hz, $^{4}J_{4,2}$= 2.3 Hz, $^{4}J_{4,6}$ = 1.7 Hz, 1H, 5-Pyrid H-4), 7.38 (m, 4H, 1-Ph H-3,4,5 and 3-Pyrid H-5), 7.36 (m, 2H, 1-Ph H-2,6), 7.25 (ddd, $^{3}J_{5,4}$ = 7.9 Hz, $^{3}J_{5,6}$ = 4.9 Hz, $^{5}J_{5,2}$= 0.9

Hz, 1H, 5-Pyrid H-5), 6.93 (s, 1H, Pyrazole H-4). ^{13}C NMR ($CDCl_3$, 100 MHz): δ (ppm) 149.5 (5-Pyrid C-6), 149.24 (5-Pyrid C-2), 149.21 (Pyrazole C-3), 148.9 (3-Pyrid C-6), 147.0 (3-Pyrid C-2), 141.4 (Pyrazole C-5), 139.3 (1-Ph C-1), 135.8 (5-Pyrid C-4), 133.2 (3-Pyrid C-4), 129.3 (1-Ph C-3,5), 128.8 (3-Pyrid C-3), 128.3 (1-Ph C-4), 126.3 (5-Pyrid C-3), 125.3 (1-Ph C-2,6), 123.7 (3-Pyrid C-5), 123.2 (5-Pyrid C-5), 105.5 (Pyrazole C-4). ^{15}N NMR ($CDCl_3$, 40 MHz): δ (ppm) –75.7 (Pyrazole N-2), –167.0 (Pyrazole N-1), 3-Pyrid N-1 and 5-Pyrid N-1 were not found. EIMS, *m/z* (rel. int.): 299 (20) $[M+H]^+$, 298 (100) $[M]^+$, 297 (86) $[M-H]^+$, 78 (20), 77 (49), 51 (35). HRMS (ESI), *m/z*: calcd. for $C_{19}H_{15}N_4^+$ 299.1291 $[M+H]^+$; found 299.1294.

5. General procedure for the preparation of 4-iodopyrazoles **3a–d**

To a solution of pyrazole **2a–d** (1.0 g) in acetic acid (10 ml), iodic acid (1.0 equiv) and iodine (0.55 equiv) were added. This mixture was stirred at 80 °C for 2 h. Then, acetic acid was distilled off, CH_2Cl_2 (20 ml) and 10% aqueous solution of Na_2CO_3 (20 ml) were added to the residue affording a basic mixture. Thereafter, a 10% aqueous solution of Na_2SO_3 (20 ml) was slowly added and the mixture was stirred to obtain a diphasic system. The organic layer was separated and the aqueous one was extracted with CH_2Cl_2 (3 × 10 ml). The combined organic phases were successively washed with a 10% aqueous solution of Na_2SO_3 (10 ml) and H_2O (20 ml) and dried over anhydrous Na_2SO_4. The solvent was evaporated under reduced pressure to obtain the crude product. This was stirred with an 1:1 mixture of EtOAc/*n*-hexane (10 ml) overnight. Then the product was filtered off and dried.

5.1. 2,2',2''-(4-Iodo-1*H*-pyrazole-1,3,5-triyl)tripyridine (**3a**)

Yield 1.065 g (75%) of a beige powder, pure according to NMR analysis; mp 113 °C. ^{1}H NMR ($CDCl_3$, 400 MHz): δ (ppm) 8.79 (m, 1H, 3-Pyrid H-6), 8.55 (ddd, $^3J_{6,5}$ = 4.9 Hz, $^4J_{6,4}$ = 1.7 Hz, $^5J_{6,3}$ = 0.9 Hz, 1H, 5-Pyrid H-6), 8.08 (ddd, $^3J_{6,5}$ = 4.8 Hz, $^4J_{6,4}$ = 1.9 Hz, $^5J_{6,3}$ = 1.2 Hz, 1H, 1-Pyrid H-6), 8.07 (m, 1H, 3-Pyrid H-3), 7.88 (m, 1H, 1-Pyrid H-3), 7.83 (m, 1H, 5-Pyrid H-4), 7.82 (m, 1H, 3-Pyrid H-4), 7.79 (m, 1H, 1-Pyrid H-4), 7.68 (ddd, $^3J_{3,4}$ = 7.8 Hz, $^4J_{3,5}$ = 1.2 Hz, $^5J_{3,6}$ = 0.9 Hz, 1H, 5-Pyrid H-3), 7.34 (ddd, $^3J_{5,4}$ = 7.5 Hz, $^3J_{5,6}$ = 4.9 Hz, $^4J_{5,3}$ = 1.1 Hz, 1H, 3-Pyrid H-5), 7.29 (ddd, $^3J_{5,4}$ = 7.6 Hz, $^3J_{5,6}$ = 4.9 Hz, $^4J_{5,3}$ = 1.2 Hz, 1H, 5-Pyrid H-5), 7.14 (ddd, $^3J_{5,4}$ = 7.4 Hz, $^3J_{5,6}$ = 4.9 Hz, $^4J_{5,3}$ = 1.1 Hz, 1H, 1-Pyrid H-5). ^{13}C NMR ($CDCl_3$, 100 MHz): δ (ppm) 152.0 (1-Pyrid C-2), 151.4 (Pyrazole C-3), 151.3 (3-Pyrid C-2), 150.3 (5-Pyrid C-2), 149.3 (5-Pyrid C-6), 149.1 (3-Pyrid C-6), 147.5 (1-Pyrid C-6), 145.1 (Pyrazole C-5), 138.4 (1-Pyrid C-4), 136.6 (3-Pyrid C-4), 136.2 (5-Pyrid C-4), 125.9 (5-Pyrid C-3), 123.22 (3-Pyrid C-5), 123.16 (5-Pyrid C-5), 123.16 (3-Pyrid C-3), 122.5 (1-Pyrid C-5), 117.4 (1-Pyrid C-3), 64.3 (Pyrazole C-4). ^{15}N NMR ($CDCl_3$, 40 MHz): δ (ppm) –66.8 (5-Pyrid N-1), –74.0 (3-Pyrid N-1), –76.7 (Pyrazole N-2), –87.7 (1-Pyrid N-1), –158.6 (Pyrazole N-1). EIMS, *m/z* (rel. int.): 425 (44) $[M]^+$, 424 (22) $[M-H]^+$, 298 (18), 270 (42), 220 (20), 81 (38), 78 (85), 69 (100), 57 (26), 51 (50), 43 (30). HRMS (ESI), *m/z*: calcd. for $C_{18}H_{13}IN_5^+$ 426.0210 $[M+H]^+$; found 426.0191.

5.2. 2,2'-(4-Iodo-1-phenyl-1*H*-pyrazole-3,5-diyl)dipyridine (**3b**)

Yield 1.180 g (83%) of a beige powder, pure according to NMR analysis; mp 147-148 °C. ^{1}H NMR ($CDCl_3$, 400 MHz): δ (ppm) 8.78 (ddd, $^3J_{6,5}$ = 4.9 Hz, $^4J_{6,4}$ = 1.8 Hz, $^5J_{6,3}$ = 0.9 Hz, 1H, 3-Pyrid H-6), 8.65 (ddd, $^3J_{6,5}$= 4.9 Hz, $^4J_{6,4}$ = 1.8 Hz, $^5J_{6,3}$ = 1.0 Hz, 1H, 5-Pyrid H-6), 8.04 (ddd, $^3J_{3,4}$ = 7.9 Hz, $^4J_{3,5}$ = 1.3 Hz, $^5J_{3,6}$ = 0.9 Hz, 1H, 3-Pyrid H-3), 7.79 (ddd, $^3J_{4,3}$ = 7.9 Hz, $^3J_{4,5}$ = 7.6 Hz, $^4J_{4,6}$ = 1.8 Hz, 1H, 3-Pyrid H-4), 7.74 (ddd, $^3J_{4,3}$ = 7.8 Hz, $^3J_{4,5}$ = 7.7 Hz, $^4J_{4,6}$ = 1.8 Hz, 1H, 5-Pyrid H-4), 7.47 (ddd, $^3J_{3,4}$ = 7.8 Hz, $^4J_{3,5}$ = 1.2 Hz, $^5J_{3,6}$ = 1.0 Hz, 1H, 5-Pyrid H-3), 7.31 (m, 3-Pyrid H-5), 7.30 (m, 2H, 1-Ph H-2,6), 7.29 (m, 5-Pyrid H-5), 7.28 (m, 2H, 1-Ph H-3,5), 7.27 (m, 1H, 1-Ph H-4). ^{13}C NMR ($CDCl_3$, 100 MHz): δ (ppm) 151.5 (3-Pyrid C-2), 151.3 (Pyrazole C-3), 149.9 (5-Pyrid C-6), 149.3 (5-Pyrid C-2), 149.2 (3-Pyrid C-6), 144.8 (Pyrazole C-5), 139.9 (1-Ph C-1), 136.4 (3-Pyrid C-3), 136.3 (5-Pyrid C-4), 128.7 (1-Ph C-3,5), 127.8 (1-Ph C-4), 126.2 (5-Pyrid C-3), 124.7 (1-Ph C-2,6), 123.5 (5-Pyrid C-5), 123.0 (3-Pyrid C-3,5), 62.6 (C-4). ^{15}N NMR ($CDCl_3$, 40 MHz): δ (ppm) –63.6 (5-Pyrid N-1), –73.0 (3-Pyrid N-1), –162.1 (Pyrazole N-1), Pyrazole N-2 was not found. EIMS, *m/z* (rel. int.): 425 (10) $[M+H]^+$, 424 (48) $[M]^+$, 423 (66) $[M-H]^+$, 296 (26), 121 (22), 111 (21), 97 (31), 86 (48), 85 (40), 84 (81), 83 (31), 81 (23), 78 (45), 77 (63), 71 (67), 69 (48), 57 (100), 55 (51), 51 (47), 43 (79), 41 (58). HRMS (ESI), *m/z*: calcd. for $C_{19}H_{14}IN_4^+$ 425.0258 $[M+H]^+$; found 425.0258.

5.3. 2-[4-Iodo-3,5-di(pyridin-3-yl)-1*H*-pyrazol-1-yl]pyridine (**3c**)

Yield 1.094 g (77%) of a yellowish powder, pure according to NMR analysis; mp 112-113 °C. ^{1}H NMR ($CDCl_3$, 400 MHz): δ (ppm) 9.22 (br s, 1H, 3-Pyrid H-2), 8.67 (br s, 1H, 3-Pyrid H-6), 8.63 (br s, 1H, 5-Pyrid H-6), 8.57 (br s, 1H, 5-Pyrid H-2), 8.27 (m, $^3J_{4,5}$ = 7.9 Hz, 1H, 3-Pyrid H-4), 8.16 (ddd, $^3J_{6,5}$ = 4.9 Hz, $^4J_{6,4}$ = 1.9 Hz, $^5J_{6,3}$ = 0.9 Hz, 1H, 1-Pyrid H-6), 7.80 (ddd, $^3J_{4,3}$= 7.9 Hz, $^3J_{4,5}$ = 7.0 Hz, $^4J_{4,6}$ = 1.9 Hz, 1H, 1-Pyrid H-4), 7.77 (m, 1H, 1-Pyrid H-3), 7.76 (m, 1H, 5-Pyrid H-4), 7.42 (dd, $^3J_{5,4}$ = 7.9 Hz, $^3J_{5,6}$= 4.9 Hz, 1H, 3-Pyrid H-5), 7.37 (dd, $^3J_{5,4}$ = 7.8 Hz, $^3J_{5,6}$ = 4.8 Hz, 1H, 5-Pyrid H-5), 7.18 (ddd, $^3J_{5,4}$ = 7.0 Hz, $^3J_{5,6}$ = 4.9 Hz, $^4J_{5,3}$= 1.5 Hz, 1H, 1-Pyrid H-5). ^{13}C NMR ($CDCl_3$, 100 MHz): δ (ppm) 151.6 (1-Pyrid C-2), 150.9 (Pyrazole C-3), 150.7 (5-Pyrid C-2), 149.6 (5-Pyrid C-6), 149.5 (3-Pyrid C-6), 149.3 (3-Pyrid C-2), 147.9 (1-Pyrid C-6), 143.2 (Pyrazole C-5), 138.6 (1-Pyrid C-4), 137.8 (5-Pyrid C-4), 135.8 (3-Pyrid C-4), 128.4 (3-Pyrid C-3), 127.4 (5-Pyrid C-3), 123.1 (broad, 3-Pyrid C-5), 122.9 (1-Pyrid C-5 and 5-Pyrid C-5), 117.2 (1-Pyrid C-3), 66.5 (Pyrazole C-4). ^{15}N NMR ($CDCl_3$, 40 MHz): δ (ppm) –87.3 (1-Pyrid N-1), Pyrazole N-1, Pyrazole N-2, 3-Pyrid N-1 and 5-Pyrid N-1 were not found. EIMS, *m/z* (rel. int.): 426 (2) $[M+H]^+$, 425 (11) $[M]^+$, 424 (28) $[M-H]^+$, 121 (100), 69 (23), 57 (21), 43 (22). HRMS (ESI), *m/z*: calcd. for $C_{18}H_{13}IN_5^+$ 426.0210 $[M+H]^+$; found 426.0213.

5.4. 3,3'-(4-Iodo-1-phenyl-1*H*-pyrazole-3,5-diyl)dipyridine (**3d**)

Yield 1.038 g (73%) of a beige powder, pure according to NMR analysis; mp 145 °C. ^{1}H NMR ($CDCl_3$, 400 MHz): δ (ppm) 9.22 (dd, $^4J_{2,4}$ = 2.2 Hz, $^5J_{2,5}$ = 0.7 Hz, 1H, 3-Pyrid H-2),

8.67 (dd, $^3J_{6,5}$ = 4.9 Hz, $^4J_{6,4}$ = 1.6 Hz, 1H, 3-Pyrid H-6), 8.64 (dd, $^3J_{6,5}$ = 4.9 Hz, $^4J_{6,4}$ = 1.7 Hz, 1H, 5-Pyrid H-6), 8.60 (dd, $^4J_{2,4}$ = 2.2 Hz, $^5J_{2,5}$ = 0.8 Hz, 1H, 5-Pyrid H-2), 8.29 (ddd, $^3J_{4,5}$ = 7.9 Hz, $^4J_{4,2}$ = 2.2 Hz, $^4J_{4,6}$ = 1.6 Hz, 1H, 3-Pyrid H-4), 7.65 (ddd, $^3J_{4,5}$ = 7.9 Hz, $^4J_{4,2}$ = 2.2 Hz, $^4J_{4,6}$ = 1.7 Hz, 1H, 5-Pyrid H-4), 7.43 (ddd, $^3J_{5,4}$ = 7.9 Hz, $^3J_{5,6}$ = 4.9 Hz, $^5J_{5,2}$ = 0.8 Hz, 1H, 3-Pyrid H-5), 7.34 (m, 1H, 5-Pyrid H-5), 7.32 (m, 3H, 1-Ph H-3,4,5), 7.25 (m, 2H, 1-Ph H-2,6). ^{13}C NMR ($CDCl_3$, 100 MHz): δ (ppm) 150.9 (5-Pyrid C-2), 150.5 (Pyrazole C-3), 150.1 (5-Pyrid C-6), 149.2 (3-Pyrid C-6), 149.0 (3-Pyrid C-2), 142.7 (Pyrazole C-5), 139.2 (1-Ph C-1), 137.7 (5-Pyrid C-4), 136.0 (3-Pyrid C-4), 129.2 (1-Ph C-3,5), 128.7 (3-Pyrid C-3), 128.4 (1-Ph C-4), 126.1 (5-Pyrid C-3), 125.0 (1-Ph C-2,6), 123.2 (3-Pyrid C-5 and 5-Pyrid C-5), 64.0 (Pyrazole C-4). ^{15}N NMR ($CDCl_3$, 40 MHz): δ (ppm) –68.5 (5-Py N-1), –69.9 (Pyrazole N-2) –74.6 (3-Py N-1), –161.9 (Pyrazole N-1). EIMS, *m/z* (rel. int.): 425 (21) $[M+H]^+$, 424 (100) $[M]^+$, 423 (46) $[M-H]^+$, 295 (21), 148 (21) 77 (69), 51 (27). HRMS (ESI), *m/z*: calcd. for $C_{19}H_{14}IN_4^+$ 425.0258 $[M+H]^+$; found 425.0259.

6. General procedure for the preparation of 4-pyrazolecarboxylic acids **4a–d**

Under argon atmosphere, a solution of the starting iodopyrazole **3a–d** (400 mg) in THF (10 ml) was cooled to −78 °C and *n*-butyllithium (1.6 M solution in hexanes, 1.1 equiv) was added in the course of 10 min. The reaction mixture was stirred at this temperature for 2–4 h (2 h in case of **3a–b** and 4 h for **3c–d**). Then CO_2 was bubbled through the mixture for 30 min at −78 °C. The reaction mixture was allowed to reach room temperature overnight and was then quenched with saturated NH_4Cl solution (10 ml). Acetic acid (0.1 ml) was added and the mixture was stirred for 30 min. Water (10 ml) was added and the mixture was exhaustively extracted with CH_2Cl_2. The combined organic phases were washed with H_2O (10 ml) and dried over anhydrous Na_2SO_4. After filtration, the solvent was evaporated under reduced pressure to obtain the crude product, which was purified by column chromatography or recrystallization (see below).

6.1. 1,3,5-Tri(pyridin-2-yl)-1*H*-pyrazole-4-carboxylic acid (**4a**)

Purification by flash chromatography (SiO_2, light petroleum/EtOAc 3:7) yielded 281 mg (87%) of a colorless solid, mp 185-186 °C. ^{1}H NMR ($CDCl_3$, 400 MHz): δ (ppm) 18.05 (br s, 1H, COOH), 8.54 (m, 1H, 5-Pyrid H-6), 8.52 (m, 1H, 5-Pyrid H-3), 8.46 (m, 1H, 3-Pyrid H-6), 8.14 (m, 1H, 1-Pyrid H-6), 7.99 (m, 1H, 5-Pyrid H-4), 7.79 (m, 1H, 1-Pyrid H-4), 7.78 (m, 1H, 3-Pyrid H-4), 7.74 (m, 1H, 3-Pyrid H-3), 7.72 (m, 1H, 1-Pyrid H-3), 7.46 (m, 1H, 5-Pyrid H-5), 7.26 (m, 1H, 3-Pyrid H-5), 7.20 (m, 1H, 1-Pyrid H-5). ^{13}C NMR ($CDCl_3$, 100 MHz): δ (ppm) 162.8 (COOH), 151.5 (1-Pyrid C-2), 150.2 (5-Pyrid C-2), 149.7 (3-Pyrid C-2), 148.6 (Pyrazole C-3), 148.5 (3-Pyrid C-6), 147.9 (1-Pyrid C-6), 146.3 (Pyrazole C-5), 145.1 (5-Pyrid C-6), 139.4 (5-Pyrid C-4), 138.4 (1-Pyrid C-4), 135.7 (3-Pyrid C-4), 127.1 (3-Pyrid C-3), 124.3 (5-Pyrid C-5), 123.22 (3-Pyrid C-5), 123.15 (1-Pyrid C-5), 122.6 (5-Pyrid C-3), 118.7 (1-Pyrid C-3), 115.6 (Pyrazole C-4). ^{15}N NMR ($CDCl_3$, 40 MHz): δ (ppm) –70.0 (3-Pyrid N-1), –78.8 (Pyrazole N-2), –84.8 (1-Pyrid N-1), –110.5 (5-Pyrid N-1), –157.8

(Pyrazole N-1). HRMS (ESI), *m/z*: calcd. for $C_{19}H_{14}N_5O_2^+$ 344.1142 [M+H]$^+$; found 344.1147.

6.2. 1-Phenyl-3,5-di(pyridin-2-yl)-1*H*-pyrazole-4-carboxylic acid (**4b**)

Purification by column chromatography (SiO_2, CH_2Cl_2/acetone 5:1) yielded 252 mg (78%) of a colorless solid, mp 194-195 °C. ^{1}H NMR ($CDCl_3$, 400 MHz): δ (ppm) 18.02 (s, 1H, COOH), 8.57 (ddd, $^3J_{6,5}$ = 4.9 Hz, $^4J_{6,4}$ = 1.8 Hz, $^5J_{6,3}$ = 1.0 Hz, 1H, 3-Pyrid H-6), 8.52 (ddd, $^3J_{6,5}$ = 5.1 Hz, $^4J_{6,4}$ = 1.7 Hz, $^5J_{6,3}$ = 0.9 Hz, 1H, 5-Pyrid H-6), 8.48 (ddd, $^3J_{3,4}$ = 8.2 Hz, $^4J_{3,5}$ = 1.2 Hz, $^5J_{3,6}$ = 0.9 Hz, 1H, 5-Pyrid H-3), 7.96 (ddd, $^3J_{4,3}$ = 8.2 Hz, $^3J_{4,5}$ = 7.6 Hz, $^4J_{4,6}$ = 1.7 Hz, 1H, 5-Pyrid H-4), 7.71 (ddd, $^3J_{4,3}$ = 7.8 Hz, $^3J_{4,5}$ = 7.6 Hz, $^4J_{4,6}$ = 1.8 Hz, 1H, 3-Pyrid H-4), 7.53 (ddd, $^3J_{3,4}$ = 7.8 Hz, $^4J_{3,5}$ = 1.2 Hz, $^5J_{3,6}$ = 1.0 Hz, 1H, 3-Pyrid H-3), 7.44 (ddd, $^3J_{5,4}$ = 7.6 Hz, $^3J_{5,6}$ = 5.1 Hz, $^4J_{5,3}$ = 1.2 Hz, 1H, 5-Pyrid H-5), 7.29 (m, 5H, 1-Ph H-2,3,4,5,6), 7.26 (ddd, $^3J_{5,4}$ = 7.6 Hz, $^3J_{5,6}$ = 4.9 Hz, $^4J_{5,3}$ = 1.2 Hz, 1H, 3-Pyrid H-5). ^{13}C NMR ($CDCl_3$, 100 MHz): δ (ppm) 162.9 (COOH), 150.4 (5-Pyrid C-2), 149.3 (3-Pyrid C-6), 149.2 (3-Pyrid C-2), 148.6 (Pyrazole C-3), 146.0 (Pyrazole C-5), 145.0 (5-Pyrid C-6), 139.3 (5-Pyrid C-4), 139.0 (Ph C-1), 135.9 (3-Pyrid C-4), 128.8 (1-Ph C-3,5), 128.3 (1-Ph C-4), 126.7 (3-Pyrid C-3), 125.0 (1-Ph C-2,6), 124.1 (5-Pyrid C-5), 123.5 (3-Pyrid C-5), 122.4 (5-Pyrid C-3), 115.1 (Pyrazole C-4). ^{15}N NMR ($CDCl_3$, 40 MHz): δ (ppm) –65.3 (3-Pyrid N-1), –75.3 (Pyrazole N-2), –110.9 (5-Pyrid N-1), –160.7 (Pyrazole N-1). HRMS (ESI), *m/z*: calcd. for $C_{20}H_{15}N_4O_2^+$ 343.1190 [M+H]$^+$; found 343.1194.

6.3. 1-(Pyridin-2-yl)-3,5-di(pyridin-3-yl)-1*H*-pyrazole-4-carboxylic acid (**4c**)

Recrystallization from EtOH gave 100 mg (31%) of colorless crystals, mp 246-252 °C. ^{1}H NMR ($CDCl_3$, 400 MHz): δ (ppm) 12.71 (br s, 1H, COOH), 8.91 (dd, $^4J_{2,4}$ = 2.2 Hz, $^4J_{2,5}$ = 0.8 Hz. 1H, 3-Pyrid H-2), 8.63 (dd, $^3J_{6,5}$ = 4.8 Hz, $^4J_{6,4}$ = 1.7 Hz, 1H, 3-Pyrid H-6), 8.55 (dd, $^3J_{6,5}$ = 4.9 Hz, $^4J_{6,4}$ = 1.7 Hz, 1H, 5-Pyrid H-6), 8.54 (dd, $^4J_{2,4}$ = 2.3 Hz, $^5J_{2,5}$ = 0.8 Hz, 1H, 5-Pyrid H-2), 8.23 (ddd, $^3J_{6,5}$ = 4.8 Hz, $^4J_{6,4}$ = 1.9 Hz, $^5J_{6,3}$ = 0.9 Hz, 1H, 1-Pyrid H-6), 8.14 (ddd, $^3J_{4,5}$ = 7.9 Hz, $^4J_{4,2}$ = 2.2 Hz, $^4J_{4,6}$ = 1.7 Hz, 1H, 3-Pyrid H-4), 8.01 (ddd, $^3J_{4,3}$ = 8.1 Hz, $^3J_{4,5}$ = 7.5 Hz, $^4J_{4,6}$ = 1.9 Hz, 1H, 1-Pyrid H-4), 7.81 (ddd, $^3J_{3,4}$ = 8.1 Hz, $^4J_{3,5}$ = 1.0 Hz, $^5J_{3,6}$ = 0.8 Hz, 1H, 1-Pyrid H-3), 7.80 (ddd, $^3J_{4,5}$ = 7.9 Hz, $^4J_{4,2}$ = 2.3 Hz, $^4J_{4,6}$ = 1.7 Hz, 1H, 5-Pyrid H-4), 7.50 (ddd, $^3J_{5,4}$ = 7.9 Hz, $^4J_{5,6}$ = 4.8 Hz, $^5J_{5,2}$ = 0.8 Hz, 1H, 3-Pyrid H-5), 7.40 (ddd, $^3J_{5,4}$ = 7.5 Hz, $^4J_{5,6}$ = 4.8 Hz, $^5J_{5,5}$ = 1.0 Hz, 1H, 1-Pyrid H-5), 7.39 (ddd, $^3J_{5,4}$ = 7.9 Hz, $^4J_{5,6}$ = 4.9 Hz, $^5J_{5,2}$ = 0.8 Hz, 1H, 5-Pyrid H-5). ^{13}C NMR ($CDCl_3$, 100 MHz): δ (ppm) 163.6 (COOH), 151.0 (1-Pyrid C-2), 150.1 (Pyrazole C-3), 150.0 (5-Pyrid C-2), 149.41 (3-Pyrid C-3), 149.37 (3-Pyrid C-6), 149.35 (5-Pyrid C-6), 148.0 (1-Pyrid C-6), 143.6 (Pyrazole C-5), 139.4 (1-Pyrid C-4), 137.7 (5-Pyrid C-4), 136.3 (3-Pyrid C-4), 128.4 (3-pyrid C-3), 126.2 (5-Pyrid C-3), 124.0 (1-Pyrid C-5), 123.1 (3-Pyrid C-5), 122.7 (5-Pyrid C-5), 119.6 (1-Pyrid C-3), 113.9 (Pyrazole C-4). ^{15}N NMR ($CDCl_3$, 40 MHz): δ (ppm) –65.9 (3-Pyrid N-1), –67.3 (5-Pyrid N-1), –81.5 (1-Pyrid N-1), Pyrazole N-1 and Pyrazole N-2 were not found. HRMS (ESI), *m/z*: calcd. for $C_{19}H_{14}N_5O_2^+$ 344.1142 [M+H]$^+$; found 344.1139.

6.4. 1-Phenyl-3,5-di(pyridin-3-yl)-1*H*-pyrazole-4-carboxylic acid (**4d**)

Purified by direct crystallization from the solution after extraction (THF/CH_2Cl_2). Yield 149 mg (46%) of yellowish crystals, mp 240-245 °C. ^{1}H NMR ($CDCl_3$, 400 MHz): δ (ppm) 12.64 (br s, 1H, COOH), 8.92 (dd, $^4J_{2,4}$ = 2.2 Hz, $^5J_{2,5}$ = 0.9 Hz, 1H, 3-Pyrid H-2), 8.62 (dd, $^3J_{6,5}$ = 4.8 Hz, $^4J_{6,4}$ = 1.7 Hz, 1H, 3-Pyrid H-6), 8.57 (m, 1H, 5-Pyrid H-2), 8.55 (m, 1H, 5-Pyrid H-6), 8.14 (ddd, $^3J_{4,5}$ = 7.9 Hz, $^4J_{4,2}$ = 2.2 Hz, $^4J_{4,6}$ = 1.7 Hz, 1H, 3-Pyrid H-4), 7.81 (ddd, $^3J_{4,5}$ = 7.9 Hz, $^4J_{4,2}$ = 2.2 Hz, $^4J_{4,6}$ = 1.7 Hz, 1H, 5-Pyrid H-4), 7.49 (ddd, $^3J_{5,4}$ = 7.9 Hz, $^3J_{5,6}$ = 4.8 Hz, $^5J_{5,2}$ = 0.9 Hz, 1H, 3-Pyrid H-5), 7.39 (m, 1H, 5-Pyrid H-5), 7.37 (m, 3H, Ph H-3,4,5), 7.35 (m, 2H, Ph H-2,6). ^{13}C NMR ($CDCl_3$, 100 MHz): δ (ppm) 163.8 (COOH), 150.5 (5-Pyrid C-2), 149.8 (Pyrazole C-3), 149.8 (5-Pyrid C-6), 149.5 (3-Pyrid C-2), 149.2 (3-Pyrid C-6), 143.8 (Pyrazole C-5), 138.4 (1-Ph C-1), 138.1 (5-Pyrid C-4), 136.6 (3-Pyrid C-4), 129.1 (1-Ph C-3,5), 128.7 (1-Ph C-4), 128.6 (3-Pyrid C-3), 125.7 (5-Pyrid C-3), 126.1 (1-Ph C-2,6), 123.1 (3-Pyrid C-5), 122.9 (5-Pyrid C-5), 112.9 (Pyrazole C-4). ^{15}N NMR ($CDCl_3$, 40 MHz): δ (ppm) –66.2 (3-Pyrid N-1), –66.3 (5-Pyrid N-1), –72.6 (Pyrazole N-2), –161.1 (Pyrazole N-1). HRMS (ESI), *m/z*: calcd. for $C_{20}H_{15}N_4O_2^+$ 343.1190 $[M+H]^+$; found 343.1190.

7. 2,2',2''-(4-Bromo-1*H*-pyrazole-1,3,5-triyl)tripyridine (**5**)

A solution of **2a** (1.076 g, 3.6 mmol) and 890 mg (5 mmol) of NBS in acetonitrile (12 ml) was heated to reflux for 2 h. After evaporation of the solvent under reduced pressure, EtOAc (60 ml) and 1 N NaOH (40 ml) were added to the remaining solid mass and the mixture was stirred until all the material had dissolved. The phases were separated and the organic phase was washed with H_2O (80 ml) and brine (80 ml) and dried over anhydrous Na_2SO_4. After filtration and evaporation of EtOAc under reduced pressure a yellowish oil was obtained, which solidified on standing. Yield 912 mg (67%), mp 42 °C. ^{1}H NMR ($CDCl_3$, 400 MHz): δ (ppm) 8.79 (m, 1H, 3-Pyrid H-6), 8.52 (m, 1H, 5-Pyrid H-6), 8.10 (m, 1H, 1-Pyrid H-6), 8.08 (m, 1H, 3-Pyrid H-3), 7.88 (m, 1H, 1-Pyrid H-3), 7.82 (m, 1H, 5-Pyrid H-4), 7.80 (m, 2H, 3-Pyrid H-4 and 1-Pyrid H-4), 7.72 (m, 1H, 5-Pyrid H-3), 7.32 (m, 1H, 3-Pyrid H-5), 7.28 (m, 1H, 5-Pyrid H-5), 7.16 (m, 1H, 1-Pyrid H-5). ^{13}C NMR ($CDCl_3$, 100 MHz): δ (ppm) 152.2 (1-Pyrid C-2), 150.8 (3-Pyrid C-2), 149.5 (3-Pyrid C-6), 149.4 (5-Pyrid C-6), 149.1 (Pyrazole C-3), 149.1 (5-Pyrid C-2), 147.6 (1-Pyrid C-6), 141.8 (Pyrazole C-5), 138.4 (1-Pyrid C-4), 136.5 (3-Pyrid C-4), 136.2 (5-Pyrid C-4), 125.5 (5-Pyrid C-3), 123.18 (3-Pyrid C-5), 123.13 (5-Pyrid C-5), 123.07 (3-Pyrid C-3), 122.5 (1-Pyrid C-5), 117.7 (1-Pyrid C-3), 96.7 (Pyrazole C-4). ^{15}N NMR ($CDCl_3$, 40 MHz): δ (ppm) –66.6 (5-Pyrid N-1), –73.8 (3-Pyrid N-1), –78.8 (Pyrazole N-2), –87.2 (1-Pyrid N-1), –162.3 (Pyrazole N-1). EIMS, *m/z* (rel. int.): 379/377 (21/24) $[M]^+$, 378/376 (44/47) $[M-H]^+$, 298 (30), 270 (32), 78 (100), 69 (60), 57 (32), 55 (41), 51 (73). HRMS (ESI), *m/z*: calcd. for $C_{18}H_{13}BrN_5^+$ 378.0349 $[M+H]^+$; found 378.0356.

8. General procedure for the Negishi cross-coupling reactions with iodopyrazoles **3a–d**

Method A: Reaction with commercially available organozinc reagents

Under argon atmosphere, Pd(dppf)Cl_2 (14.6 mg, 0.02 mmol) and CuI (7.6 mg, 0.04 mmol) were added to a solution of starting iodopyrazole **3a–d** (425 mg resp. 424 mg, 1 mmol) in dry THF (20 ml). Then, the solution of the corresponding organozinc bromide (4 ml, 2 mmol, 0.5 M in THF) was added via syringe. The reaction mixture was refluxed for 15–24 h under argon atmosphere. After cooling to rt the reaction was quenched with saturated aqueous NH_4Cl solution (10 ml), H_2O was added (10 ml), and the product was exhaustively extracted with CH_2Cl_2 (30 ml). The combined organic layers were washed with 10% aqueous ammonia (2 × 20 ml) and water (2 × 20 ml). After drying over anhydrous Na_2SO_4 and subsequent filtration, the solvents were evaporated under reduced pressure to obtain the crude product which was purified by column chromatography or recrystallization.

Method B: Reaction with in situ generated organozinc reagents

Under argon atmosphere, to the solution of the corresponding organolithium reagent (3 mmol, 1 M 2-thienyllithium in THF/hexanes or 1 M (trimethylsilyl)methyllithium in pentane or 1.9 M phenyllithium in dibutylether) in dry THF (20 ml) at –78 °C, zinc chloride (3 ml, 3 mmol, 1M solution in Et_2O) was added and the mixture was stirred at this temperature for 10 min. Then the mixture was allowed to reach rt during 1 h. To this mixture the starting iodopyrazole **3a–d** (0.425 g, 1 mmol), Pd(dppf)Cl_2 (14.6 mg, 0.02 mmol) and CuI (7.6 mg, 0.04 mmol) were added and the reaction mixture was refluxed for 15–24 h under argon atmosphere. After cooling to rt the reaction was quenched with saturated aqueous NH_4Cl solution (10 ml), H_2O was added (10 ml), and the product was exhaustively extracted with dichloromethane (30 ml). The combined organic layers were washed with 10% aqueous ammonia (2 × 20 ml) and water (2 × 20 ml), and dried over anhydrous Na_2SO_4. After filtration, the solvents were evaporated under reduced pressure to obtain the crude product which was purified by column chromatography or recrystallization.

8.1. 2,2',2''-(4-Phenyl-1*H*-pyrazole-1,3,5-triyl)tripyridine (**6a**)

Prepared via method A with 1.5 equiv of phenylzinc bromide. After column chromatography (SiO_2, CH_2Cl_2/acetone 5:1) a colorless solid was obtained. Yield 71 mg (19%), mp 123-125 °C. ^1H NMR ($CDCl_3$, 400 MHz): δ (ppm) 8.64 (ddd, $^3J_{6,5}$ = 4.9 Hz, $^4J_{6,4}$ = 1.8 Hz, $^5J_{6,3}$ = 1.0 Hz, 1H, 3-Pyrid H-6), 8.45 (m, 1H, 5-Pyrid H-6), 8.09 (ddd, $^3J_{6,5}$ = 4.9 Hz, $^4J_{6,4}$ = 1.9 Hz, $^5J_{6,3}$ = 0.9 Hz, 1H, 1-Pyrid H-6), 7.93 (ddd, $^3J_{3,4}$ = 8.2 Hz, $^4J_{3,5}$ = 1.0 Hz, $^5J_{3,6}$ = 0.9 Hz, 1H, 1-Pyrid H-3), 7.78 (ddd, $^3J_{4,3}$ = 8.2 Hz, $^3J_{4,5}$ = 7.4 Hz, $^4J_{4,6}$ = 1.9 Hz, 1H, 1-Pyrid H-4), 7.54 (ddd, $^3J_{4,3}$ = 8.0 Hz, $^3J_{4,5}$ = 7.5 Hz, $^4J_{4,6}$ = 1.8 Hz, 1H, 3-Pyrid H-4), 7.51 (ddd, $^3J_{4,3}$ = 7.9 Hz, $^3J_{4,5}$ = 7.6 Hz, $^4J_{4,6}$ = 1.8 Hz, 1H, 5-Pyrid H-4), 7.37 (ddd, $^3J_{3,4}$ = 8.0 Hz, $^4J_{3,5}$ = 1.2 Hz, $^5J_{3,6}$ = 1.0 Hz, 1H, 3-Pyrid H-3), 7.22 (m, 1H, 4-Ph H-4), 7.21 (m, 4H, 4-Ph H-2,3,5,6), 7.17 (ddd, $^3J_{5,4}$ = 7.5 Hz, $^3J_{5,6}$ = 4.9 Hz, $^4J_{5,3}$ = 1.2 Hz, 1H, 3-Pyrid H-5), 7.13 (m, 2H, 1-Pyrid H-5, 5-Pyrid H-3), 7.12 (m, 1H, 5-Pyrid H-5). ^{13}C NMR ($CDCl_3$, 100 MHz): δ (ppm) 152.5 (1-Pyrid C-2), 151.9 (3-Pyrid C-2), 150.3 (5-Pyrid C-2), 150.0 (Pyrazole C-3), 149.7 (3-Pyrid C-6), 149.0 (5-Pyrid C-6), 147.4 (1-Pyrid C-6), 140.9 (Pyrazole C-5), 138.1 (1-Pyrid C-4), 135.9

(3-Pyrid C-4), 135.8 (5-Pyrid C-4), 132.2 (4-Ph C-1), 130.6 (4-Ph C-2,6), 128.0 (4-Ph C-3,5), 127.0 (4-Ph C-4), 125.2 (5-Pyrid C-3), 123.5 (3-Pyrid C-3), 123.1 (Pyrazole C-4), 122.5 (3-Pyrid C-5), 122.4 (5-Pyrid C-5), 122.1 (1-Pyrid C-5), 118.3 (1-Pyrid C-3). ^{15}N NMR ($CDCl_3$, 40 MHz): δ (ppm) –66.0 (5-Pyrid N-1), –71.2 (3-Pyrid N-1), –80.1 (Pyrazole N-2), –86.5 (1-Pyrid N-1), –162.7 (Pyrazole N-1). HRMS (ESI), *m/z*: calcd. for $C_{24}H_{18}N_5^+$ 376.1557 $[M+H]^+$; found 376.1558.

8.2. 2,2'-(1,4-Diphenyl-1*H*-pyrazole-3,5-diyl)dipyridine (**6b**)

Prepared via method A. After column chromatography (SiO_2, CH_2Cl_2/acetone 10:1) a colorless solid was obtained. Yield 101 mg (27%), mp 125-130 °C. ^{1}H NMR ($CDCl_3$, 400 MHz): δ (ppm) 8.64 (ddd, $^3J_{6,5}$ = 4.9 Hz, $^4J_{6,4}$ = 1.8 Hz, $^5J_{6,3}$ = 1.0 Hz, 1H, 3-Pyrid H-6), 8.52 (ddd, $^3J_{6,5}$ = 4.9 Hz, $^4J_{6,4}$ = 1.8 Hz, $^5J_{6,3}$ = 1.0 Hz, 1H, 5-Pyrid H-6), 7.55 (ddd, $^3J_{4,3}$ = 7.9 Hz, $^3J_{4,5}$ = 7.6 Hz, $^4J_{4,6}$ = 1.8 Hz, 1H, 3-Pyrid H-4), 7.50 (ddd, $^3J_{4,3}$ = 7.8 Hz, $^3J_{4,5}$ = 7.7 Hz, $^4J_{4,6}$ = 1.8 Hz, 1H, 5-Pyrid H-4), 7.38 (ddd, $^3J_{3,4}$ = 7.9 Hz, $^4J_{3,5}$ = 1.2 Hz, $^5J_{3,6}$ = 1.0 Hz, 1H, 3-Pyrid H-3), 7.36 (m, 2H, 1-Ph H-2,6), 7.28 (m, 2H, 1-Ph H-3,5), 7.26 (m, 1H, 1-Ph H-4), 7.21 (m, 5H, 4-Ph H-2,3,4,5,6), 7.17 (ddd, $^3J_{5,4}$ = 7.6 Hz, $^3J_{5,6}$ = 4.9 Hz, $^4J_{5,3}$ = 1.2 Hz, 1H, 3-Pyrid H-5), 7.14 (ddd, $^3J_{5,4}$ = 7.7 Hz, $^3J_{5,6}$ = 4.9 Hz, $^4J_{5,3}$ = 1.2 Hz, 5-Pyrid H-5), 7.05 (ddd, $^3J_{3,4}$ = 7.8 Hz, $^4J_{3,5}$ = 1.2 Hz, $^5J_{3,6}$ = 1.0 Hz, 5-Pyrid H-3). ^{13}C NMR ($CDCl_3$, 100 MHz): δ (ppm) 152.2 (3-Pyrid C-2), 149.7 (3-Pyrid C-6), 149.65 (5-Pyrid C-2), 149.6 (5-Pyrid C-6), 149.5 (Pyrazole C-3), 140.7 (Pyrazole C-5), 140.0 (1-Ph C-1), 136.0 (5-Pyrid C-4), 135.9 (3-Pyrid C-4), 132.4 (4-Ph C-1), 130.5 (4-Ph C-2,6), 128.5 (1-Ph C-3,5), 128.0 (4-Ph C-3,5), 127.3 (1-Ph C-4), 126.8 (4-Ph C-4), 125.9 (5-Pyrid C-3), 123.4 (3-Pyrid C-3), 122.8 (5-Pyrid C-5), 122.4 (Pyrazole C-4), 122.3 (3-Pyrid C-5). ^{15}N NMR ($CDCl_3$, 40 MHz): δ (ppm) –63.4 (5-Pyrid N-1), –71.3 (3-Pyrid N-1), –76.6 (Pyrazole N-2), –165.8 (Pyrazole N-1). HRMS (ESI), *m/z*: calcd. for $C_{25}H_{19}N_4^+$ 375.1604 $[M+H]^+$; found 375.1605.

8.3. 1,1',3,3',5,5'-Hexa(pyridin-2-yl)-1*H*,1'*H*-4,4'-bipyrazole (**7a**)

Prepared via method B. Compound **7a** was isolated as a colorless powder *via* column chromatography (SiO_2, *n*-hexane/EtOAc 1:1). Yield 197 mg (66%), mp 108-110 °C. ^{1}H NMR ($CDCl_3$, 400 MHz): δ (ppm) 8.51 (m, 1H, 3-Pyrid H-6), 8.17 (m, 1H, 5-Pyrid H-6), 8.04 (m, 1H, 1-Pyrid H-6), 7.93 (m, 1H, 1-Pyrid H-3), 7.76 (m, 1H, 1-Pyrid H-4), 7.64 (m, 1H, 3-Pyrid H-3), 7.51 (m, 1H, 3-Pyrid H-4), 7.48 (m, 1H, 5-Pyrid H-4), 7.15 (m, 1H, 5-Pyrid H-3), 7.09 (m, 1H, 1-Pyrid H-5), 7.07 (m, 1H, 3-Pyrid H-5), 7.00 (m, 1H, 5-Pyrid H-5). ^{13}C NMR ($CDCl_3$, 100 MHz): δ (ppm) 152.4 (1-Pyrid C-2), 151.5 (3-Pyrid C-2), 151.3 (Pyrazole C-3), 149.69 (5-Pyrid C-2), 149.66 (3-Pyrid C-6), 148.6 (5-Pyrid C-6), 147.3 (1-Pyrid C-6), 142.2 (Pyrazole C-5), 138.2 (1-Pyrid C-4), 136.19 (5-Pyrid C-4), 136.16 (3-Pyrid C-4), 124.5 (5-Pyrid C-3), 122.4 (3-Pyrid C-5), 122.3 (5-Pyrid C-5), 122.3 (3-Pyrid C-3), 122.0 (1-Pyrid C-5), 118.0 (1-Pyrid C-3), 113.3 (Pyrazole C-4). ^{15}N NMR ($CDCl_3$, 40 MHz): δ (ppm) –67.8 (5-Pyrid N-1), –72.2 (3-Pyrid N-1), –80.1 (Pyrazole N-2), –88.1 (1-Pyrid N-1), –162.6 (Pyrazole N-1). HRMS (ESI), *m/z*: calcd. for $C_{36}H_{25}N_{10}^+$ 597.2258 $[M+H]^+$; found 597.2264.

8.4 1,1'-Diphenyl-3,3',5,5'-tetra(pyridin-2-yl)-1*H*,1'*H*-4,4'-bipyrazole (**7b**)

Prepared via method A. Compound **7b** was isolated as beige crystals *via* column chromatography (SiO_2, CH_2Cl_2/acetone 10:1 → 3:1) in the course of the reaction of **3b** with PhZnCl (see 8.2.). Yield 143 mg (48%), mp 95-97 °C. ^{1}H NMR ($CDCl_3$, 400 MHz): δ (ppm) 8.47 (ddd, $^3J_{6,5}$ = 4.8 Hz, $^4J_{6,4}$ = 1.8 Hz, $^5J_{6,3}$ = 1.0 Hz, 1H, 3-Pyrid H-6), 8.23 (ddd, $^3J_{6,5}$ = 4.9 Hz, $^4J_{6,4}$ = 1.8 Hz, $^5J_{6,3}$ = 1.0 Hz, 1H, 5-Pyrid H-6), 7.59 (ddd, $^3J_{3,4}$ = 8.0 Hz, $^4J_{3,5}$ = 1.2 Hz, $^5J_{3,6}$ = 1.0 Hz, 1H, 3-Pyrid H-3), 7.48 (ddd, $^3J_{4,3}$ = 8.0 Hz, $^3J_{4,5}$ = 7.5 Hz, $^4J_{4,6}$ = 1.8 Hz, 1H, 3-Pyrid H-4), 7.41 (ddd, $^3J_{4,3}$ = 7.9 Hz, $^3J_{4,5}$ = 7.6 Hz, $^4J_{4,6}$ = 1.8 Hz, 1H, 5-Pyrid H-4), 7.30 (m, 2H, 1-Ph H-2,6), 7.24 (m, 2H, 1-Ph H-3,5), 7.21 (m, 1H, 1-Ph H-4), 7.06 (ddd, $^3J_{3,4}$ = 7.9 Hz, $^4J_{3,5}$ = 1.2 Hz, $^5J_{3,6}$ = 1.0 Hz, 1H, 5-Pyrid H-3), 7.04 (ddd, $^3J_{5,4}$ = 7.5 Hz, $^3J_{5,6}$ = 4.9 Hz, $^4J_{5,3}$ = 1.3 Hz, 1H, 3-Pyrid H-5), 6.99 (ddd, $^3J_{5,4}$ = 7.6 Hz, $^3J_{5,6}$ = 4.8 Hz, $^4J_{5,3}$ = 1.2 Hz, 1H, 5-Pyrid H-5). ^{13}C NMR ($CDCl_3$, 100 MHz): δ (ppm) 151.9 (3-Pyrid C-2), 150.6 (Pyrazole C-3), 149.5 (3-Pyrid C-6), 149.2 (5-Pyrid C-6), 149.1 (5-Pyrid C-2), 141.7 (Pyrazole C-5), 140.1 (1-Ph C-1), 136.1 (5-Pyrid C-4), 136.0 (3-Pyrid C-4), 128.5 (1-Ph C-3,5), 127.0 (1-Ph C-4), 125.1 (5-Pyrid C-3), 124.7 (1-Ph C-2,6), 122.5 (5-Pyrid C-5), 122.10 (3-Pyrid C-5), 122.08 (3-Pyrid C-3), 112.8 (Pyrazole C-4). ^{15}N NMR ($CDCl_3$, 40 MHz): δ (ppm) –65.7 (5-Pyrid N-1), –72.4 (3-Pyrid N-1), –76.5 (Pyrazole N-2), –166.0 (Pyrazole N-1). HRMS (ESI), *m/z*: calcd. for $C_{38}H_{27}N_8^+$ 595.2353 [M+H]$^+$; found 595.2357.

8.5. 2,2',2'',2'''-(1*H*-Pyrazole-1,3,4,5-tetrayl)tetrapyridine (**9a**)

Prepared via method A. After column chromatography (SiO_2, $CHCl_3$/MeOH 10:1) a colorless solid was obtained. Yield 290 mg (77%), mp 197-199 °C. ^{1}H NMR ($CDCl_3$, 400 MHz): δ (ppm) 8.53 (m, 1H, 4-Pyrid H-6), 8.52 (m, 1H, 3-Pyrid H-6), 8.43 (m, 1H, 5-Pyrid H-6), 8.13 (m, 1H, 1-Pyrid H-6), 7.89 (m, 1H, 1-Pyrid H-3), 7.79 (m, 1H, 1-Pyrid H-4), 7.66 (m, 1H, 3-Pyrid H-3), 7.62 (m, 1H, 3-Pyrid H-4), 7.55 (m, 1H, 5-Pyrid H-4), 7.52 (m, 1H, 4-Pyrid H-4), 7.34 (m, 1H, 5-Pyrid H-3), 7.22 (m, 1H, 4-Pyrid H-3), 7.17 (m, 1H, 3-Pyrid H-5), 7.14 (m, 2H, 1-Pyrid H-5 and 5-Pyrid H-5), 7.12 (4-Pyrid H-5). ^{13}C NMR ($CDCl_3$, 100 MHz): δ (ppm) 152.51 (4-Pyrid C-2), 152.48 (1-Pyrid C-2), 151.9 (3-Pyrid C-2), 150.4 (Pyrazole C-3), 150.1 (5-Pyrid C-2), 149.4 (3-Pyrid C-6), 149.2 (4-Pyrid C-6), 149.0 (5-Pyrid C-6), 147.6 (1-Pyrid C-6), 141.9 (Pyrazole C-5), 138.2 (1-Pyrid C-4), 136.0 (3-Pyrid C-4), 135.7 (5-Pyrid C-4), 135.6 (4-Pyrid C-4), 125.9 (4-Pyrid C-3), 125.6 (5-Pyrid C-3), 123.2 (3-Pyrid C-3), 123.0 (Pyrazole C-4), 122.5 (3-Pyrid C-5), 122.5 (5-Pyrid C-5), 122.2 (1-Pyrid C-5), 121.7 (4-Pyrid C-5), 118.4 (1-Pyrid C-3). ^{15}N NMR ($CDCl_3$, 40 MHz): δ (ppm) –66.5 (5-Pyrid N-1), –65.4 (4-Pyrid N-1), –70.9 (3-Pyrid N-1), –86.4 (1-Pyrid N-1), –80.3 (Pyrazole N-2), –163.1 (Pyrazole N-1). HRMS (ESI), *m/z*: calcd. for $C_{23}H_{17}N_6^+$ 377.1509 [M+H]$^+$; found 377.1514.

8.6. 2,2',2''-(1-Phenyl-1*H*-pyrazole-3,4,5-triyl)tripyridine (**9b**)

Prepared via method A. After column chromatography (SiO_2, CH_2Cl_2/acetone 5:1) a colorless solid was obtained. Yield 319 mg (85%), mp 183-184 °C. ^{1}H NMR ($CDCl_3$, 400 MHz): δ

(ppm) 8.52 (ddd, $^3J_{6,5}$ = 4.9 Hz, $^4J_{6,4}$ = 1.8 Hz, $^5J_{6,3}$ = 1.1 Hz, 1H, 3-Pyrid H-6), 8.51 (ddd, $^3J_{6,5}$ = 4.9 Hz, $^4J_{6,4}$ = 1.9 Hz, $^5J_{6,3}$ = 1.0 Hz, 1H, 4-Pyrid H-6), 8.48 (ddd, $^3J_{6,5}$ = 4.9 Hz, $^4J_{6,4}$ = 1.8 Hz, $^5J_{6,3}$ = 1.0 Hz, 1H, 5-Pyrid H-6), 7.66 (ddd, $^3J_{3,4}$ = 7.9 Hz, $^4J_{3,5}$ = 1.5 Hz, $^5J_{3,6}$ = 1.1 Hz, 1H, 3-Pyrid H-3), 7.61 (ddd, $^3J_{4,3}$ = 7.9 Hz, $^3J_{4,5}$ = 7.2 Hz, $^4J_{4,6}$ = 1.8 Hz, 1H, 3-Pyrid H-4), 7.53 (ddd, $^3J_{4,3}$ = 7.8 Hz, $^3J_{4,5}$ = 7.5 Hz, $^4J_{4,6}$ = 1.9 Hz, 1H, 4-Pyrid H-4), 7.53 (ddd, $^3J_{4,3}$ = 7.8 Hz, $^3J_{4,5}$ = 7.6 Hz, $^4J_{4,6}$ = 1.8 Hz, 1H, 5-Pyrid H-4), 7.37 (m, 2H, 1-Ph H-2,6), 7.30 (m, 2H, 1-Ph H-3,5), 7.28 (m, 1H, 1-Ph H-4), 7.27 (ddd, $^3J_{3,4}$ = 7.8 Hz, $^4J_{3,5}$ = 1.2 Hz, $^5J_{3,6}$ = 1.0 Hz, 1H, 4-Pyrid H-3), 7.26 (ddd, $^3J_{3,4}$ = 7.8 Hz, $^4J_{3,5}$ = 1.2 Hz, $^5J_{3,6}$ = 1.0 Hz, 1H, 5-Pyrid H-3), 7.16 (ddd, $^3J_{5,4}$ = 7.2 Hz, $^3J_{5,6}$ = 4.9 Hz, $^4J_{5,3}$ = 1.5 Hz, 1H, 3-Pyrid H-5), 7.14 (ddd, $^3J_{5,4}$ = 7.6 Hz, $^3J_{5,6}$ = 4.9 Hz, $^4J_{5,3}$ = 1.2 Hz, 1H, 5-Pyrid H-5), 7.12 (ddd, $^3J_{5,4}$ = 7.5 Hz, $^3J_{5,6}$ = 4.9 Hz, $^4J_{5,3}$ = 1.2 Hz, 1H, 4-Pyrid H-5). ^{13}C NMR ($CDCl_3$, 100 MHz): δ (ppm) 152.8 (4-Pyrid C-2), 152.1 (3-Pyrid C-2), 149.9 (Pyrazole C-3), 149.5 (5-Pyrid C-6), 149.41 (5-Pyrid C-2), 149.39 (3-Pyrid C-6), 149.1 (4-Pyrid C-6), 141.8 (Pyrazole C-5), 140.0 (1-Ph C-1), 136.0 (3-Pyrid C-4), 135.9 (5-Pyrid C-4), 135.6 (4-Pyrid C-4), 128.7 (1-Ph C-3,5), 127.5 (1-Ph C-4), 126.0 (5-Pyrid C-3), 125.9 (4-Pyrid C-3), 125.3 (1-Ph C-2,6), 123.1 (3-Pyrid C-3), 122.8 (5-Pyrid C-5), 122.4 (3-Pyrid C-5), 122.3 (Pyrazole C-4), 121.6 (4-Pyrid C-5). ^{15}N NMR ($CDCl_3$, 40 MHz): δ (ppm) –63.9 (5-Pyrid N-1), –65.8 (4-Pyrid N-1), –70.9 (3-Pyrid N-1), –76.5 (Pyrazole N-2), –166.1 (Pyrazole N-1). HRMS (ESI), *m/z*: calcd. for $C_{24}H_{18}N_5^+$ 376.1557 $[M+H]^+$; found 376.1558.

8.7. 2,2',2''-[4-(Thiophen-2-yl)-1*H*-pyrazole-1,3,5-triyl]tripyridine (**10a**)

Prepared via method B. Recrystallization from EtOH afforded colorless crystals. Yield 332 mg (87%), mp 122-124 °C. ^{1}H NMR ($CDCl_3$, 400 MHz): δ (ppm) 8.70 (m, 1H, 3-Pyrid H-6), 8.49 (m, 1H, 5-Pyrid H-6), 8.09 (m, 1H, 1-Pyrid H-6), 7.93 (m, 1H, 1-Pyrid H-3), 7.79 (m, 1H, 1-Pyrid H-4), 7.63 (m, 1H, 3-Pyrid H-4), 7.61 (m, 1H, 5-Pyrid H-4), 7.51 (m, 1H, 3-Pyrid H-3), 7.33 (m, 1H, 5-Pyrid H-3), 7.23 (m, 2H, 3-Pyrid H-5 and Th H-5), 7.19 (m, 1H, 5-Pyrid H-5), 7.14 (m, 1H, 1-Pyrid H-5), 6.93 (m, 1H, Th H-4), 6.92 (m, Th H-3). ^{13}C NMR ($CDCl_3$, 100 MHz): δ (ppm) 152.4 (1-Pyrid C-2), 151.7 (3-Pyrid C-2), 150.6 (Pyrazole C-3), 150.0 (5-Pyrid C-2), 149.7 (3-Pyrid C-6), 149.1 (5-Pyrid C-6), 147.5 (1-Pyrid C-6), 141.7 (Pyrazole C-5), 138.2 (1-Pyrid C-4), 136.0 (3-Pyrid C-4), 135.9 (5-Pyrid C-4), 132.8 (Th C-2), 128.6 (Th C-3), 126.8 (Th C-4), 126.1 (Th C-5), 125.4 (5-Pyrid C-3), 123.5 (3-Pyrid C-3), 122.8 (3-Pyrid C-5), 122.7 (5-Pyrid C-5), 122.2 (1-Pyrid C-5), 118.2 (1-Pyrid C-3), 116.0 (Pyrazole C-4). ^{15}N NMR ($CDCl_3$, 40 MHz): δ (ppm) –66.0 (5-Pyrid N-1), –70.8 (3-Pyrid N-1), –80.2 (Pyrazole N-2), –86.9 (1-Pyrid N-1), –162.0 (Pyrazole N-1). EIMS, *m/z* (rel. int.): 381 (9) $[M]^+$, 380 (15) $[M-H]^+$, 299 (41), 298 (67), 221 (14), 167 (16), 150 (13), 149 (77), 111 (21), 97 (25), 85 (26), 83 (31), 78 (30), 71 (65), 70 (29), 69 (57), 67 (22), 57 (100), 56 (21). HRMS (ESI), *m/z*: calcd. for $C_{22}H_{16}N_5S^+$ 382.1121 $[M+H]^+$; found 382.1126.

8.8. 2,2'-[1-Phenyl-4-(thiophen-2-yl)-1*H*-pyrazole-3,5-diyl]dipyridine (**10b**)

Prepared via method B. After column chromatography (SiO_2, CH_2Cl_2/acetone 10:1) a colorless solid was obtained. Yield 224 mg (59%), mp 154-156 °C. ^{1}H NMR ($CDCl_3$,

400 MHz): δ (ppm) 8.69 (ddd, $^3J_{6,5}$ = 4.9 Hz, $^4J_{6,4}$ = 1.8 Hz, $^5J_{6,3}$ = 1.0 Hz, 1H, 3-Pyrid H-6), 8.57 (m, 1H, 5-Pyrid H-6), 7.62 (ddd, $^3J_{4,3}$ = 7.9 Hz, $^3J_{4,5}$ = 7.5 Hz, $^4J_{4,6}$ = 1.8 Hz, 1H, 3-Pyrid H-4), 7.57 (m, 1H, 5-Pyrid H-4), 7.51 (ddd, $^3J_{3,4}$ = 7.9 Hz, $^4J_{3,5}$ = 1.3 Hz, $^5J_{3,6}$ = 1.0 Hz, 1H, 3-Pyrid H-3), 7.36 (m, 2H, 1-Ph H-2,6), 7.28 (m, 2H, 1-Ph H-3,5), 7.26 (m, 1H, 1-Ph H-4), 7.21 17 (ddd, $^3J_{5,4}$ = 7.5 Hz, $^3J_{5,6}$ = 4.9 Hz, $^4J_{5,3}$ = 1.3 Hz, 1H, 3-Pyrid H-5), 7.20 (m, 2H, 5-Pyrid H-3 and Th H-5), 7.19 (5-Pyrid H-5), 6.92 (m, 1H, Th H-3), 6.91 (m, 1H, Th H-4). ^{13}C NMR ($CDCl_3$, 100 MHz): δ (ppm) 151.9 (3-Pyrid C-2), 150.1 (Pyrazole C-3), 149.7 (5-Pyrid C-6), 149.6 (3-Pyrid C-6), 149.3 (5-Pyrid C-2), 141.4 (Pyrazole C-5), 139.9 (1-Ph C-1), 136.1 (5-Pyrid C-4), 136.0 (3-Pyrid C-4), 133.1 (Th C-2), 128.6 (1-Ph C-3,5), 128.3 (Th C-3), 127.5 (1-Ph C-4), 126.8 (Th C-4), 125.9 (Th C-5), 125.9 (5-Pyrid C-3), 125.1 (1-Ph C-2,6), 123.5 (3-Pyrid C-3), 123.1 (5-Pyrid C-5), 122.6 (3-Pyrid C-5), 115.2 (Pyrazole C-4). ^{15}N NMR ($CDCl_3$, 40 MHz): δ (ppm) –63.0 (5-Pyrid N-1), –70.6 (3-Pyrid N-1), –76.6 (Pyrazole N-2), –165.4 (Pyrazole N-1). HRMS (ESI), *m/z*: calcd. for $C_{23}H_{17}N_4S^+$ 381.1168 $[M+H]^+$; found 381.1167.

8.9. 2,2',2"-{4-[(Trimethylsilyl)methyl]-1*H*-pyrazole-1,3,5-triyl}tripyridine (**11a**)

Prepared via method B. The crude product was digested with *n*-hexane/CH_2Cl_2 8:2 to afford a pale yellow solid. Yield 293 mg (73%), mp 152-153 °C. ^{1}H NMR ($CDCl_3$, 400 MHz): δ (ppm) 8.66 (m, 1H, 3-Pyrid H-6), 8.59 (m, 1H, 5-Pyrid H-6), 8.12 (m, 1H, 1-Pyrid H-6), 8.09 (m, 1H, 3-Pyrid H-3), 7.77 (1-Pyrid H-3), 7.75 (m, 2H, 3-Pyrid H-4 and 1-Pyrid H-4), 7.73 (5-Pyrid H-4), 7.42 (m, 1H, 5-Pyrid H-3), 7.22 (m, 2H, 3-Pyrid H-5 and 5-Pyrid H-5), 7.09 (1-Pyrid H-5), 2.68 (s, 2H, $SiCH_2$), –0.33 (s, 9H, $SiMe_3$). ^{13}C NMR ($CDCl_3$, 100 MHz): δ (ppm) 153.8 (3-Pyrid C-2), 152.5 (1-Pyrid C-2), 151.3 (5-Pyrid C-2), 149.6 (Pyrazole C-3), 149.1 (5-Pyrid C-6), 148.4 (3-Pyrid C-6), 147.9 (1-Pyrid C-6), 139.0 (Pyrazole C-5), 138.0 (1-Pyrid C-4), 136.4 (3-Pyrid C-4), 136.1 (5-Pyrid C-4), 125.2 (5-Pyrid C-3), 122.2 (5-Pyrid C-5), 122.2 (3-Pyrid C-3), 122.1 (3-Pyrid C-5), 121.6 (Pyrazole C-4), 121.5 (1-Pyrid C-5), 117.4 (1-Pyrid C-3), 12.6 ($SiCH_2$), –1.4 ($SiMe_3$). ^{15}N NMR ($CDCl_3$, 40 MHz): δ (ppm) –66.0 (5-Pyrid N-1), –71.9 (3-Pyrid N-1), –81.9 (Pyrazole N-2), –88.4 (1-Pyrid N-1), –166.0 (Pyrazole N-1). HRMS (ESI), *m/z*: calcd. for $C_{22}H_{24}N_5Si^+$ 386.1795 $[M+H]^+$; found 386.1802.

8.10. 2,2'-{1-Phenyl-4-[(trimethylsilyl)methyl]-1*H*-pyrazole-3,5-diyl}dipyridine (**11b**)

Prepared via method B. Recrystallization from *n*-hexane/CH_2Cl_2 afforded brownish crystals. Yield 327 mg (85%), mp 135-138 °C. ^{1}H NMR ($CDCl_3$, 400 MHz): δ (ppm) 8.65 (m, 1H, 5-Pyrid H-6), 8.64 (m, 1H, 3-Pyrid H-6), 8.04 (m, 1H, 3-Pyrid H-3), 7.71 (m, 1H, 3-Pyrid H-4), 7.62 (m, 1H, 5-Pyrid H-4), 7.30 (m, 2H, 1-Ph H-2,6), 7.28 (m, 2H, 1-Ph H-3,5), 7.21 (m, 1H, 1-Ph H-4), 7.20 (m, 2H, 5-Pyrid H-3 and 5-Pyrid H-5), 7.18 (m, 1H, 3-Pyrid H-5), 2.76 (s, 2H, $SiCH_2$), –0.33 (s, 9H, $SiMe_3$). ^{13}C NMR ($CDCl_3$, 100 MHz): δ (ppm) 154.2 (3-Pyrid C-2), 150.8 (5-Pyrid C-2), 149.8 (5-Pyrid C-6), 149.1 (Pyrazole C-3), 148.6 (3-Pyrid C-6), 140.3 (1-Ph C-1), 139.0 (Pyrazole C-5), 136.05 (3-Pyrid C-4), 136.01 (5-Pyrid C-4), 128.7 (1-Ph C-3,5), 126.8 (1-Ph C-4), 125.5 (5-Pyrid C-3), 124.6 (1-Ph C-2,6), 122.3 (5-Pyrid C-5), 121.9

(3-Pyrid C-3), 121.8 (3-Pyrid C-5), 120.7 (Pyrazole C-4), 12.5 ($SiCH_2$), –1.5 ($SiMe_3$). HRMS (ESI), *m/z*: calcd. for $C_{23}H_{25}N_4Si^+$ 385.1843 $[M+H]^+$; found 385.1845.

8.11. 5-Phenyl-1,3-di(pyridin-2-yl)-1*H*-pyrazolo[3,4-*a*]quinolizin-6-ium iodide (**12**)

According to method B, from **3a** with (phenylethynyl)zinc chloride under standard conditions and after column chromatography (SiO_2, CH_2Cl_2/MeOH 10:1) compound **12** was isolated as brownish crystals in 14% yield (74 mg), mp 310-315 °C. ^1H NMR ($CDCl_3$, 400 MHz): δ (ppm) 9.16 (s, 1H, H-4), 9.03 (m, 1H, H-7), 8.76 (m, 1H, H-10), 8.73 (m, 1H, 1-Pyrid H-6), 8.70 (m 1H, 3-Pyrid H-6), 8.54 (m, 1H, H-9), 8.31 (m, 1H, 3-Pyrid H-3), 8.19 (m, 2H, 1-Pyrid H-3,4), 8.15 (m, 1H, H-8), 7.85 (m, 1H, 3-Pyrid H-4), 7.77 (m, 2H, Ph H-2,6), 7.70 (m, 3H, Ph H-3,4,5), 7.67 (m, 1H, 1-Pyrid H-5), 7.37 (m, 1H, 3-Pyrid H-5). ^{13}C NMR ($CDCl_3$, 100 MHz): δ (ppm) 151.8 (1-Pyrid C-2), 150.5 (3-Pyrid C-2), 149.5 (3-Pyrid C-6), 149.0 (1-Pyrid C-6), 146.5 (C-3), 140.4 (1-Pyrid C-4), 140.0 (C-5), 138.9 (C-9), 137.0 (3-Pyrid C-4), 135.4 (C-7), 134.4 (C-10a), 132.8 (C-10b), 131.8 (Ph C-1), 131.3 (Ph C-4), 130.4 (Ph C-2,6), 130.3 (Ph C-3,5), 125.6 (1-Pyrid C-5), 125.08 (C-8), 125.05 (C-10), 124.3 (3-Pyrid C-5), 123.3 (C-3a), 121.6 (3-Pyrid C-3), 121.5 (C-4), 121.4 (1-Pyrid C-3). ^{15}N NMR ($CDCl_3$, 40 MHz): δ (ppm) –73.8 (3-Pyrid N-1), –85.7 (1-Pyrid N-1), –95.5 (1-Pyrid N-1), –174.0 (Pyrazole N-1), –176.2 (N-6), Pyrazole N-2 was not found. HRMS, *m/z*: calcd. for $C_{26}H_{18}N_5^+$ 400.1551 $[M]^+$; found 400.1563.

9. Attempted Feringa cross-coupling with **3a**

9.1. 2-[3,5-Di(pyridin-2-yl)-1*H*-pyrazol-1-yl]-6-phenylpyridine (**8**)

According to ref. [4], under argon atmosphere, to a solution of starting iodopyrazole **3a** (0.20 g, 0.47 mmol) in dry toluene (3.1 ml) $Pd[P(t\text{-}Bu)_3]_2$ (12 mg, 0.023 mmol) was added. Then the solution of phenyllithium (0.63 ml, 1.2 mmol, 1.9 M in dibutylether) in THF (1.37 ml) was prepared. This 0.6 M solution of phenyllithium (1.2 ml, 0.72 mmol) was slowly added over 70 min using a syringe pump. After the addition was completed, the reaction mixture was stirred 90 min at rt and quenched with saturated aqueous NH_4Cl solution (10 ml). The product was exhaustively extracted with CH_2Cl_2 (20 ml) and the organic layer was washed with water (20 ml). After drying over anhydrous Na_2SO_4 and filtration, the solvents were evaporated under reduced pressure to obtain the crude product. Separation by column chromatography (SiO_2, CH_2Cl_2/acetone 5:1) gave new product **8** as a yellowish, amorphous mass. Yield 28 mg (16%). ^1H NMR ($CDCl_3$, 400 MHz): δ (ppm) 8.69 (m, 1H, 3-Pyrid H-6), 8.53 (m, 1H, 5-Pyrid H-6), 8.15 (m, 1H, 3-Pyrid H-3), 7.96 (m, 1H, 1-Pyrid H-3), 7.90 (m, 1H, 1-Pyrid H-4), 7.79 (m, 1H, 5-Pyrid H-4), 7.77 (m, 1H, 3-Pyrid H-4), 7.64 (m, 1H, 1-Pyrid H-5), 7.63 (m, 1H, 5-Pyrid H-3), 7.30 (m, 2H, Ph H-2,6), 7.29 (m, 1H, Ph H-4), 7.27 (m, 1H, 5-Pyrid H-5), 7.26 (m, 1H, 3-Pyrid H-5), 7.23 (m, 2H, Ph H-3,5). ^{13}C NMR ($CDCl_3$, 100 MHz): δ (ppm) 154.7 (1-Pyrid C-6), 152.5 (Pyrazole C-3), 152.1 (1-Pyrid C-2), 151.7 (3-Pyrid C-2), 151.6 (5-Pyrid C-2), 149.5 (3-Pyrid C-6), 149.3 (5-Pyrid C-6), 144.2 (Pyrazole C-5), 139.2 (1-Pyrid C-4), 137.8 (Ph C-1), 136.3 (3-Pyrid C-4), 136.3 (5-Pyrid C-4), 129.0 (Ph C-4), 128.3 (Ph C-3,5), 126.4 (Ph C-2,6), 123.8 (5-Pyrid C-3), 122.9 (3-Pyrid C-5), 122.4 (5-

Pyrid C-5), 120.4 (3-Pyrid C-3), 117.9 (1-Pyrid C-5), 115.2 (1-Pyrid C-3), 108.7 (Pyrazole C-4). ^{15}N NMR ($CDCl_3$, 40 MHz): δ (ppm) –69.6 (5-Pyrid N-1), –77.7 (3-Pyrid N-1), –79.8 (Pyrazole N-2), –95.5 (1-Pyrid N-1), –162.5 (Pyrazole N-1). HRMS (ESI), *m/z*: calcd. for $C_{24}H_{18}N_5^+$ 376.1557 $[M+H]^+$; found 376.1555.

References

1. Andrews, P. C.; Deacon, G. B.; Frank, R.; Fraser, B. H.: Junk, P. C.; MacLellan, J. G.; Massi, M.; Moubaraki, B.; Murray, K. S.; Silberstein, M. *Eur. J. Inorg. Chem.* **2009**: 744-751. doi:10.1002/ejic.200801006
2. Ferles, M.; Kafka, S.; Šilhánková, A.; Šputová, M. *Collect. Czech. Chem. Commun.* **1981**, *46*, 1167-1172. doi.org/10.1135/cccc19811167
3. Ferles, M.; Liboska, R.; Trška, P. *Collect. Czech. Chem. Commun.* **1990**, *55*, 1228-1233. doi.org/10.1135/cccc19901228
4. Giannerini, M.; Fañanás-Mastral, M; Feringa, B. L. *Nat Chem.* **2013**, *5*, 667-672. doi. 10.1038/nchem.1678

X-ray analysis of compound **12**

The X-ray intensity data was measured on Bruker D8 Venture diffractometer equipped with multilayer monochromators, Mo K/a INCOATEC micro focus sealed tube and Kryoflex II cooling device. The structure was solved by *charge flipping* and refined by *full-matrix least-squares techniques*. Non-hydrogen atoms were refined with *anisotropic displacement parameters*. Hydrogen atoms were inserted at calculated positions and refined with a riding model. The following software was used: *Bruker SAINT software package*[i] using a narrow-frame algorithm for frame integration, *SADABS*[ii] for absorption correction, *OLEX2*[iii] for structure solution, refinement molecular diagrams and graphical user-interface, *SHELXL-2013*[iv] for refinement and graphical user-interface, *Platon*[v] for symmetry check and Cg(Pi-Ring) interactions calculations. Experimental data and CCDC-code can be found in Table S1. Crystal data, data collection parameters, and structure refinement details are given in Tables S2 and S3. The molecular structure in “Ortep View” is displayed in Figure S1. Packing and plane stacking is shown in Figure S2.

Table S1: Experimental parameter and CCDC-Code.

**Sample**	**Machine**	**Source**	**Temp.**	**Detector Distance**	**Time/ Frame**	**#Frames**	**Frame width**	**CCDC**
	Bruker		[K]	[mm]	[s]		[°]	
**12**	D8	Mo	100	34	8	924	0.8	1528377

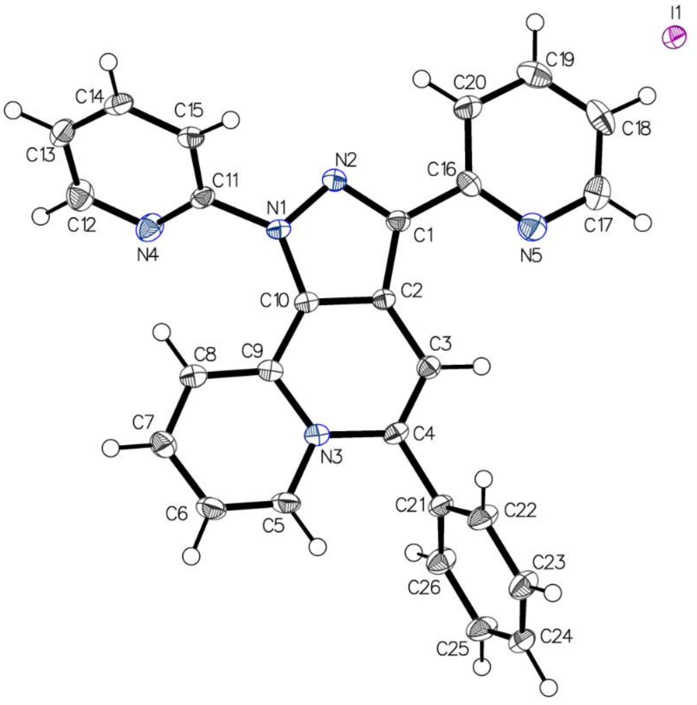

Figure S1: Asymmetric Unit of compound **12**, drawn with 50% displacement ellipsoids. Solvent omitted for clarity. Bond precision: C-C=0.0031A.

Table S2: Sample and crystal data of compound **12**.

**Chemical formula**	C28H20Cl6IN5	**Crystal system**	triclinic	
**Formula weight [g/mol]**	766.09	**Space group**	*P-1*	
**Temperature [K]**	100	**Z**	2	
**Measurement method**	\Φ and \ω scans	**Volume [Å^3]**	1516.71(11)	
**Radiation (Wavelength [Å])**	MoKα (λ = 0.71073)	**Unit cell dimensions [Å] and [°]**	11.0386(4)	97.0055(19)
**Crystal size / [mm^3]**	0.259 × 0.13 × 0.038		12.3012(5)	111.8606(17)
**Crystal habit**	clear yellow plate		13.7210(6)	112.6868(18)
**Density (calculated) / [g/cm^3]**	1.677	**Absorption coefficient / [mm^{-1}]**	1.613	
**Abs. correction Tmin**	0.6721	**Abs. correction Tmax**	0.746	
**Abs. correction type**	multi-scan	**F(000) [e$^-$]**	756	

Table S3 Data collection and structure refinement of compound **12**.

**Index ranges**	-15 ≤ h ≤ 15, -17 ≤ k ≤ 17, -19 ≤ l ≤ 19	**Theta range for data collection [°]**	4.892 to 60.17	
**Reflections number**	47670	**Data / restraints / parameters**	8886/0/361	
**Refinement method**	Least squares	**Final R indices**	all data	R1 = 0.0432, wR2 = 0.0588
**Function minimized**	Σ w(F_o^2 - F_c^2)^2		I>2σ(I)	R1 = 0.0271, wR2 = 0.0552
**Goodness-of-fit on F^2**	1.033	**Weighting scheme**	w=1/[σ^2(F_o^2)+(0.0254P)^2+0.6335P]	
**Largest diff. peak and hole [e Å^{-3}]**	0.99/-0.68		where P=(F_o^2+2F_c^2)/3	

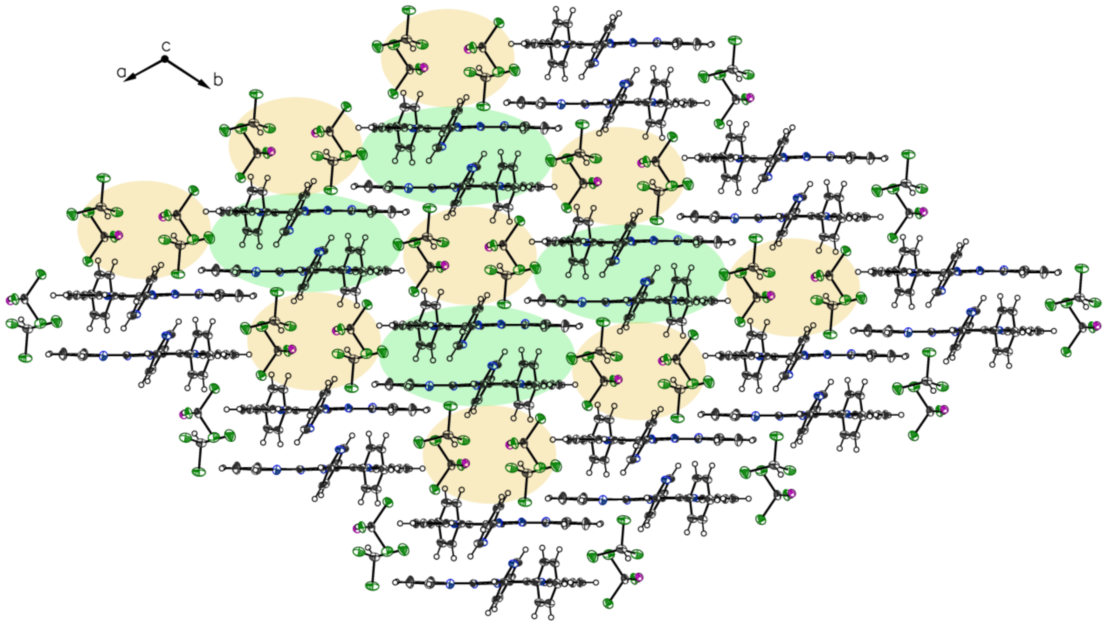

Figure S2: Packing of **12**. The layered system is influenced by π plane interactions (light green shaded). The light orange shaded area highlights the solvent and iodo channels along axis c.

References for X-ray analysis

i Bruker SAINT v8.32BA Copyright © 2005-2016 Bruker AXS

ii Sheldrick, G. M. (1996). *SADABS*. University of Göttingen, Germany.

iii Dolomanov, O. V.; Bourhis, L. J.; Gildea, R. J; Howard, J. A. K.; Puschmann, H.; OLEX2, *J. Appl. Cryst*. **2009**, *42*, 339-341.

iv Sheldrick, G. M. (1996). *SHELXL*. University of Göttingen, Germany.

v Spek, A. L. *Acta Cryst.* **2009**, *D65*, 148-155.
